# Systematic review and meta-analysis of interventions for mental health in extreme weather events

**DOI:** 10.1136/bmjgh-2025-020407

**Published:** 2026-05-28

**Authors:** Jai K Das, Ayesha Arshad Ali, Anzal Taufeeq Jangda, Nisma Riaz, Zulfiqar A Bhutta

**Affiliations:** 1 Institute for Global Health and Development, The Aga Khan University, Karachi, Pakistan; 2 Department of Pediatrics and Child Health, The Aga Khan University, Karachi, Pakistan; 3 Morristown Medical Center, Morristown, New Jersey, USA; 4 Profit by Pakistan Today, Karachi, Pakistan; 5 Centre for Global Child Health, The Hospital for Sick Children, Toronto, Ontario, Canada

**Keywords:** Systematic review, Environmental health, Mental Health & Psychiatry

## Abstract

**Introduction:**

Climate change leading to extreme weather events (EWEs) poses critical threat to human health, including mental well-being. As the effects of climate change are becoming more pronounced, there is a need to identify effective interventions to mitigate the mental health impacts.

**Methods:**

The aim of this review was to synthesise the existing literature on interventions to prevent and manage mental health symptoms in people affected by EWEs. We conducted a comprehensive literature search in MEDLINE, EBSCO CINAHL and Cochrane till 7 February 2024 and in Web of Science and EMBASE till 20 February 2024. Randomised controlled trials (RCTs), quasi-experimental studies and before-after study designs were included. Our search yielded 11 083 potentially relevant papers, which were screened by titles and full texts and 33 studies were finally included in the review. We recorded the study, participants, intervention, disaster and other relevant data on Excel in duplicate. We conducted a meta-analysis separately for intervention-control and before-after studies using Review Manager 5.4. The Cochrane Risk of Bias tool II was used to assess risk of bias in RCTs and the risk of bias in non-randomised studies of interventions tool for quasi-experimental and before-after designs. Main outcomes were symptoms of post-traumatic stress disorder (PTSD), depression, anxiety, stress, general functioning and well-being.

**Results:**

There was significant improvement in mental health symptoms following implementation of various interventions, leading to a decrease in PTSD symptoms (standardised mean difference (SMD) −2.92, 95% CI (−4.53 to −1.31), six intervention-control studies and SMD −0.71, 95% CI (−0.95 to −0.48), five before-after studies), depression symptoms (SMD −0.28, 95% CI (−0.53 to −0.03), three intervention-control studies and SMD −1.00, 95% CI (−1.41 to −0.59), 11 before-after studies), anxiety symptoms (SMD −0.38, 95% CI (−0.69 to −0.06), two studies and SMD −0.35, 95% CI (−0.47 to −0.23), eight before-after studies), stress symptoms (SMD −0.82, 95% CI (−1.10 to −0.53), two before-after studies), general functioning impairment (SMD −1.22, 95% CI (−1.73 to −0.70), three before-after studies) and an improvement in well-being (SMD 0.29, 95% CI (0.15 to 0.42), three intervention-control studies and SMD 0.67, 95% CI (0.25 to 1.09), two before-after studies).

**Conclusion:**

There are existing interventions to improve mental health after an EWE, and these should be integrated into all relevant disaster management plans.

**WHAT IS ALREADY KNOWN ON THIS TOPIC**

- Extreme weather events (EWEs) like floods, heatwaves and wildfires are becoming more severe and frequent due to climate change, posing not only physical but also serious psychological risks, such as post-traumatic stress disorder (PTSD), depression, increased suicide rates, etc. While emerging interventions like cognitive-behavioural therapy, mindfulness and community-based support show promise in reducing mental health symptoms, mental health response plans remain inconsistent and underdeveloped.

**WHAT THIS STUDY ADDS**

- In this systematic review, we found a significant reduction in PTSD, depression, anxiety, stress and general functioning impairment symptoms and improved well-being of the study participants affected by EWEs after implementation of various mental health-focused interventions.

**HOW THIS STUDY MIGHT AFFECT RESEARCH, PRACTICE OR POLICY**

- Existing disaster guidelines focus more on physical health, and there’s a need for tailored, evidence-based mental health strategies in EWE-affected areas. This review assesses multiple existing interventions and provides evidence to develop and implement tailored strategies to manage mental health symptoms after EWEs.

## Introduction

 Anthropogenic greenhouse gas emissions have raised global average temperatures by around 1.5°C since the late 19th century,^1^ and heatwaves have become more frequent, prolonged and intense.^2^ Extreme weather events (EWEs), including floods, wildfires, hurricanes and heatwaves, have increased in severity and frequency due to climate change.^1^ These events pose significant threats to infrastructure, public health and economic stability, and the effects of these are not just physically devastating but also have profound psychological impacts, which are often overshadowed by the focus on physical infrastructure and health consequences.^2^ The vulnerable populations of women, children and economically disadvantaged individuals are specifically at a higher risk of adverse outcomes due to climate change-related disasters.^2 3^

Disruption of existing physical and social systems due to climate change and EWEs can indirectly affect mental health,^4^ and these mental health impacts can be particularly severe among vulnerable populations such as children, older people, low-income communities and indigenous people.^5^ According to numerous studies, the escalation of EWE has exacerbated mental health issues in children and adolescents, involving cases of post-traumatic stress disorder (PTSD), depression, anxiety and similar effects noted within older adults.^6^,^8^ Several systematic reviews have been conducted to evaluate the effect of different EWEs on mental health, such as hurricane and flood exposure and their association with PTSD,^3 9^ heatwaves with increased aggression and suicide rates, although the mechanism for this is unclear.^4^

As the effects of climate change and EWEs become more pronounced, there is an increasing need to identify effective interventions that can mitigate the subsequent mental health impacts,^10^ and interventions aimed at mitigating the psychological effects of EWEs are emerging as essential components of comprehensive disaster management strategies. Different types of interventions have been proposed, from individual-level strategies such as cognitive-based interventions and mindfulness-based interventions to community-based interventions, such as social support groups and ecotherapy programmes.^2^ These interventions have shown the potential to reduce the negative mental health symptoms and build resilience to future impacts, and there is evidence to suggest that social support, community resilience and environmental factors can all play important roles in reducing the mental health impacts.^11^

However, unlike the well-established guidelines for managing physical health emergencies during and after EWEs, including the protocols for injuries, infections and chronic disease management, mental health response remains poorly defined and inconsistent. The Intergovernmental Panel on Climate Change report on managing the risks of extreme events and disasters to advance climate change adaptation from 2012 mentions the provision of mental health services along with other basic necessities, such as safe water, sufficient and safe shelter, etc; however, tailored plans or protocols are lacking.^12^ Gaps in disaster preparedness plans have been highlighted in several published reviews, particularly regarding the inclusion of mental health strategies, but the mental health response frameworks often lack the granularity required to implement concrete actions and hence address specific needs.^13 14^ There is insufficient quantitative evidence focusing on the relationship between EWEs and the effectiveness of various interventions for mental health, which may contribute to the evidence for effective interventions combating mental health issues.^15^

Considering the projected increase in EWEs, there is a need for effective mental health interventions and guidelines tailored specifically to post-EWE scenarios and to gauge the sustained impact of these, especially in frequently affected areas. The aim of this systematic review is to synthesise the existing literature on interventions to prevent and manage adverse mental health symptoms, specifically after EWEs and provide an understanding of the effectiveness of different interventions.

## Methods

The Cochrane Handbook^16^ guidelines were followed to conduct the systematic review, and Preferred Reporting Items for Systematic Reviews and Meta-Analyses (PRISMA) guidelines^17^ were used to report the findings.

### Inclusion criteria

We included randomised controlled trials (RCTs), quasi-experimental studies and before-after study designs. These study designs were preferred due to their ability to establish stronger relationships between intervention and mental health outcomes, as they comprised a comparison data, which can help assess the actual effectiveness of the intervention in EWE.

This systematic review included studies that assessed the interventions for both the prevention and the management of mental health symptoms and well-being after EWEs in population settings. We included all populations, including children, women and adults, and the EWE comprised temperature extremes, heavy precipitation and pluvial floods, river floods, droughts, storms (including tropical cyclones, tornadoes and hurricanes) and wildfires/bushfires, compound events (multivariate and concurrent extremes).^1^ Studies were included if they had implemented and tested an intervention with an objective to prevent or manage the mental health of the study population, and these interventions could comprise Mental Health and Psychosocial Support (MHPSS) interventions,^18^ psychotherapy, yoga and community rebuilding/resilience interventions. However, any studies focusing solely on physical health were excluded from the review. The outcomes of interest included symptoms of depression, anxiety, PTSD, stress, suicidal ideation, substance abuse, general functioning impairment and general well-being (self-reported symptoms which did not necessarily fit any criteria). The definitions and assessment scales were as defined by the authors or by consulting an expert in the team.

### Literature search

We formulated a search strategy using medical subject headings (MeSH) and keywords relating to climate change and mental health in MEDLINE, EBSCO CINAHL and Cochrane Library (online supplemental files S1–S5) till 7 February 2024 and in Web of Science and EMBASE till 20 February 2024. We used a human filter, but no publication year, publication status or outcomes restrictions were applied. We also searched relevant systematic reviews and bibliographies of included studies to identify any missing papers.

### Screening

The results from the search of all databases were exported to EndNote and uploaded into Covidence.^19^ In the first phase, two reviewers screened study titles and abstracts independently, and then full texts were screened in duplicate. Disagreements were resolved through discussion or consulting a third reviewer. If full texts of studies were not available online or if further clarification or data were required, the authors of those papers were contacted for assistance.

### Data extraction

Key variables were extracted by two reviewers independently and recorded in standardised data collection forms. The data collected included study details (journal, publication year, design and location), EWE (event, scope/scale and year), participants (age, number and inclusion criteria), intervention details (description, duration, frequency and delivery), outcomes (description/definition of outcomes, time points, method of assessment and mental health symptom scores) and study limitations.

### Assessment of risk of bias

The Cochrane Risk of Bias tool II (ROB2)^20^ was used to assess risk of bias in RCTs and Risk of Bias in Non-randomised Studies of Interventions (ROBINS)^21^ for quasi-experimental and before-after designs by two reviewers. ROB2 assessed trials on the following domains: randomisation process (D1), deviations from the intended interventions (D2), missing outcome data (D3), measurement of the outcome (D4) and selection of the reported result (D5). ROBINS assessed studies for the following domains: bias due to confounding (D1), selection of participants (D2), classification of interventions (D3), deviations from intended interventions (D4), missing data (D5), measurement of outcomes (D6) and selection of reported results (D7).

### Data synthesis

We conducted a meta-analysis on Review Manager (RevMan) software V.5.4.1.^22^ Standardised mean difference (SMD), along with a 95% CI, was calculated for each outcome using mean scores and SD of symptoms for each outcome. Intervention-control studies and before-after studies were analysed separately. We used SMDs instead of mean differences, as studies used different scales to assess the outcomes. We performed a random effects analysis for all comparisons due to variations between the studies in terms of settings, population and intervention, using the inverse-variance method for continuous outcomes. We assessed statistical heterogeneity using τ^2^, I^2^ and the significance of the χ^2^ test.^16^ Symptoms related to PTSD, depression, anxiety, stress, general functioning and well-being were the outcomes analysed for each group.

### Subgroup analysis

Subgroup analysis was done depending on the data availability for age groups (children/adults), socioeconomic status (low- and middle-income countries (LMIC)/high-income countries (HIC)), disaster type and mental health assessment symptom scales. We also planned to do a subgroup analysis by individual or group-based counselling and hospital or community settings, but there was a lot of variability between studies, and hence, it could not be done.

## Results

**Figure 1:**
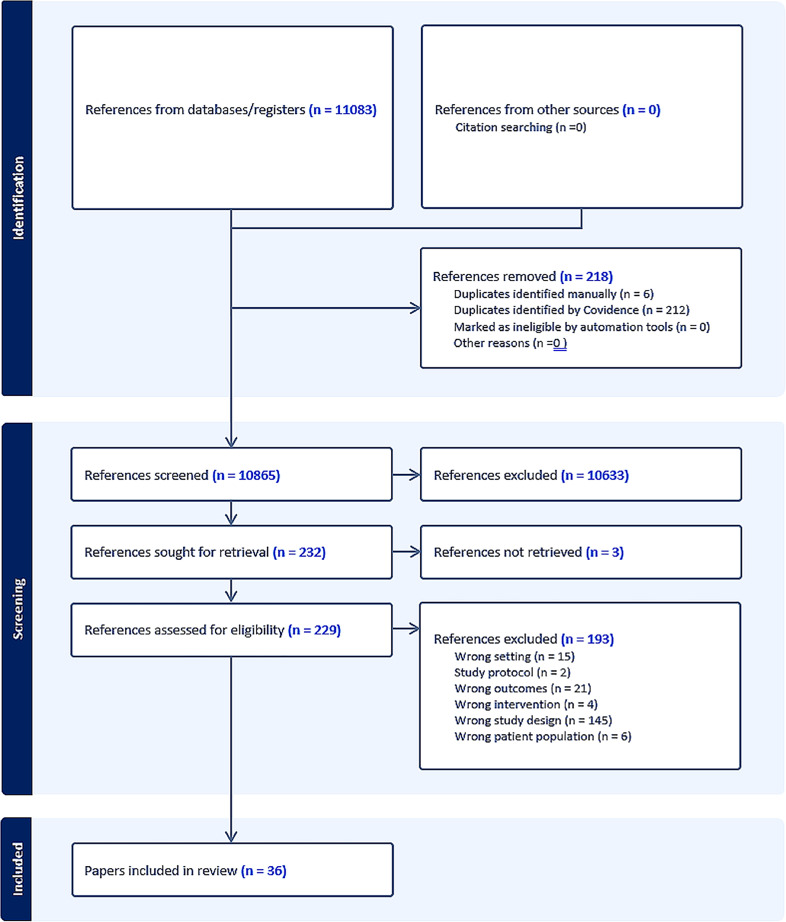
Preferred Reporting Items for Systematic Reviews and Meta-Analyses.

The search identified 11 083 potentially relevant studies; after deduplication, 10 865 titles and abstracts were screened. A total of 232 full texts were reviewed for relevance, and 36 papers from 33 primary studies were included^23^,^58^ (figure 1 and online supplemental table 1).

**Table 1:** Subgroup estimates

Subgroups	Post-traumatic stress disorder	Depression	Anxiety	Stress	General functioning	Well-being
World Bank income level classification						
LMIC—intervention/control	SMD −9.05, 95% CI (−26.74 to 8.63), two studies	SMD −0.10, 95% CI (−0.26 to 0.06), one study	SMD −0.13, 95% CI (−0.97 to 0.71), one study		SMD −0.09, 95% CI (−0.32 to 0.13), two studies	
HIC—intervention/control	SMD −0.30, 95% CI (−0.49 to −0.10), four studies	SMD of −0.41, 95% CI (−0.64 to −0.18), two studies	SMD −0.42, 95% CI (−0.76 to −0.08), one study	(SMD −0.18, 95% CI (−0.40 to 0.03), three studies	SMD −0.58, 95% CI (−0.92 to −0.24), one study	SMD 0.29, 95% CI (0.15 to 0.42), three studies
LMIC—before/after		SMD −4.76, 95% CI (−5.55 to −3.97), one study	SMD −0.13, 95% CI (−0.97 to 0.71), one study			SMD 0.88, 95% CI (0.58 to 1.17), one study
HIC—before/after	SMD −0.71, 95% CI (−0.95 to −0.48) five studies	SMD −0.72, 95% CI (−0.96 to −0.47), 10 studies	SMD −0.35, 95% CI (−0.47 to −0.23), seven studies	SMD −0.82, 95% CI (−1.10 to −0.53), two studies	SMD −1.22, 95% CI (−1.73 to −0.70), three studies	SMD 0.45, 95% CI (0.11 to 0.78), one study
Age						
Adults—intervention/control	SMD −3.55, 95% CI (−5.47 to −1.63), five studies	SMD −0.28, 95% CI (−0.53 to −0.03), three studies	SMD −0.38, 95% CI (−0.69 to −0.06), two studies	SMD −0.14, 95% CI (−0.48 to 0.19), two studies	SMD −0.27, 95% CI (−0.62 to 0.08), three studies	SMD 0.33, 95% CI (0.18 to 0.47), two studies
Children—intervention/control	SMD −0.07, 95% CI (−0.54 to 0.40), one study			SMD −0.24, 95% CI (−0.62 to 0.14), one study		SMD 0.02, 95% CI (−0.36 to 0.40), one study
Adults—before/after	SMD −0.90, 95% CI (−1.34 to −0.46), three studies	SMD −1.11, 95% CI (−1.92 to −0.30), six studies	SMD −0.33, 95% CI (−0.47 to −0.19), six studies	SMD −0.82, 95% CI (−1.10 to −0.53), two studies	SMD −1.50, 95% CI (−1.93 to −1.07), two studies	SMD 0.67, 95% CI (0.25 to 1.09), two studies
Children—before/after	SMD: −0.63, 95% CI (−0.89 to −0.37), two studies	SMD −0.94, 95% CI (−1.37 to −0.51), five studies	SMD −0.41, 95% CI (−0.66 to −0.16), two studies		SMD −0.86, 95% CI (−1.11 to −0.60), one study	
Extreme weather event						
Hurricane—intervention/control	SMD −0.17, 95% CI (−0.43 to 0.09), two studies	SMD −0.32, 95% CI (−0.64 to 0.00), one study		SMD −0.14, 95% CI (−0.48 to 0.19), two studies		SMD 0.19, 95% CI (−0.37 to 0.74), one study
Flood—intervention/control	SMD −9.05, 95% CI (−26.74 to 8.63), two studies	SMD −0.10, 95% CI (−0.26 to 0.06), one study	SMD −0.13, 95% CI (−0.97 to 0.71), one study		SMD −0.09, 95% CI (−0.32 to 0.13), two studies	SMD 0.34, 95% CI (0.19 to 0.49), one study
Wildfire/bushfire— intervention/control	SMD −0.46, 95% CI (−0.75 to −0.16), two studies	SMD −0.52, 95% CI (−0.86 to −0.17), one study	SMD −0.42, 95% CI (−0.76 to −0.08), one study		SMD −0.58, 95% CI (−0.92 to −0.24), one study	
Tornado— intervention/control				SMD −0.24, 95% CI (−0.62 to 0.14), one study		SMD 0.02, 95% CI (−0.36 to 0.40), one study
Hurricane—before/after	SMD −0.60, 95% CI (−0.80 to −0.40), three studies	SMD −0.75, 95% CI (−1.03 to −0.47), eight studies	SMD −0.33, 95% CI (−0.46 to −0.20), five studies		SMD −0.86, 95% CI (−1.11 to −0.60), one study	SMD 0.45, 95% CI (0.11 to 0.78), one study
Wildfire/Bushfire— before/after	SMD −1.42, 95% CI (−2.25 to −0.59), one study	SMD −0.49, 95% CI (−1.01 to 0.04), one study	SMD −0.48, 95% CI (−1.04 to 0.09), one study	SMD −0.82, 95% CI (−1.10 to −0.53), two studies	SMD −1.50, 95% CI (−1.93 to −1.07), two studies	
Flood—before/after		SMD −4.76, 95% CI (−5.55 to −3.97), one study	SMD −0.13, 95% CI (−0.97 to 0.71), one study			
Typhoon— before/after						SMD 0.88, 95% CI (0.58 to 1.17), one study
Tornado—before/after	SMD −0.83, 95% CI (−1.48 to −0.18), one study	SMD −0.65, 95% CI (−1.29 to −0.01), one study	SMD −0.77, 95% CI (−1.42 to −0.13), one study			

HIC, high-income countries; LMIC, low- and middle-income countries; SMD, standardised mean difference.

### Characteristics of included studies

A total of 33 intervention studies from 36 papers were included in this review; 16 were intervention-control (eight^28 29 36 37 39 44 46 53 54 57^ RCTs and eight quasi-experimental^24^,^2732 38 49 55^), while 17^2330 31 33^,^35 40^ were before-after study designs. 25 studies were conducted in high- and upper-middle-income countries and the rest in LMIC. Children and adolescents under the age of 18 were included in six studies, while other studies only included adults. The EWEs included wildfires or bushfires in four studies,^23 41 53 54 57^ floods in eight studies,^25 26 36 37 40 46 55 58^ cyclones in one study,^27^ hurricanes in 17 studies,^2430^,^32 34 35 38 39 42^ tornadoes in two studies^28 29 49^ and typhoons in one study.^33^ No studies were found on extreme heat.

All mental health interventions occurred in a post-disaster setting and included behavioural therapy sessions, narrative counselling, psychoeducation, yoga and breathing exercises and mindfulness training. One study had a nutrition intervention (B complex and broad-spectrum vitamins compared with vitamin D) to improve mental health.^37^ Interventions in 10 studies were conducted in camps or crisis centres or community settings,^25 33 35 38 40 41 45 46 48 51 52^ five were online,^4453^,^55 57^ four were in hospital settings^23 32 38 43^ (three at outpatient centres and one inpatient), six studies implemented school-based interventions,^24 31 34 42 49 56^ and the rest did not define it clearly. Trained healthcare workers (general physicians, psychiatrists, therapists, etc) delivered the intervention in 17 studies,^2325 26 30^,^32 34 35 38 39 42 48^ licensed professionals or trained social workers in five studies^41 43 45 56 57^ and volunteers, including schoolteachers, in four studies.^24 27 40 57^ The duration of the intervention ranged from one session only to regular sessions spanning several months. Participants were recruited through invitation in some studies, while others were screened in certain affected areas, such as camps or schools and included participants based on symptoms at the time of screening and willingness to participate. Mental health symptoms such as PTSD, depressive symptoms, etc, were evaluated using different scales by teachers, volunteers or therapists and participants who were assessed to be very sick were referred to or excluded in most studies.

### Quality assessment

Assessment was the same for all outcomes. Overall, one study was at low risk of bias, and five studies were at moderate risk of bias or had some concerns, while all other studies were at high/serious risk of bias. ROB2 assessed one study at low risk of bias; one had some concerns overall, and eight were at high risk of bias (online supplemental figure 1). The ROBINS tool assessed four studies at moderate risk of bias and all others at serious risk of bias (online supplemental figure 2).

### Effect estimates

The pooled effect estimates for various outcomes are reported below. We did not pool Kaplan^37^ as it was a nutrition intervention and it suggested comparable PTSD symptoms in both groups, significant reduction in depression (SMD –0.68, 95% CI (−1.15 to −0.20), one study), anxiety (SMD −3.08, 95% CI (−5.75 to −0.40) one study) and stress (SMD −0.56, 95% CI (−1.03 to −0.09), one study) (online supplemental figures 3–6).

#### Post-traumatic stress disorder

**Figure 2:**
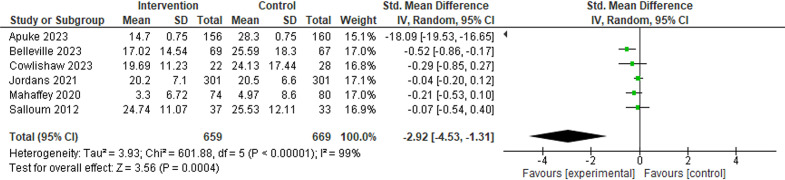
Post-traumatic stress disorder symptoms (intervention-control studies).

Six intervention-control studies and seven before-after studies reported PTSD symptoms. Analysis of intervention-control studies suggested a significant decrease in PTSD symptoms in the intervention group compared with the control (SMD −2.92, 95% CI (−4.53 to −1.31), six studies). In before-after studies, PTSD symptoms reduced after the intervention (SMD −0.71, 95% CI (−0.95 to −0.48), five studies). Subgroup analysis of PTSD symptoms suggested a significant reduction in PTSD symptoms in HICs, adults and children (before-after studies only), in wildfires (intervention-control studies only), bushfires, hurricanes and tornadoes (before-after studies) (figure 2, online supplemental figures 7–15 and table 1).

#### Depression

**Figure 3:**
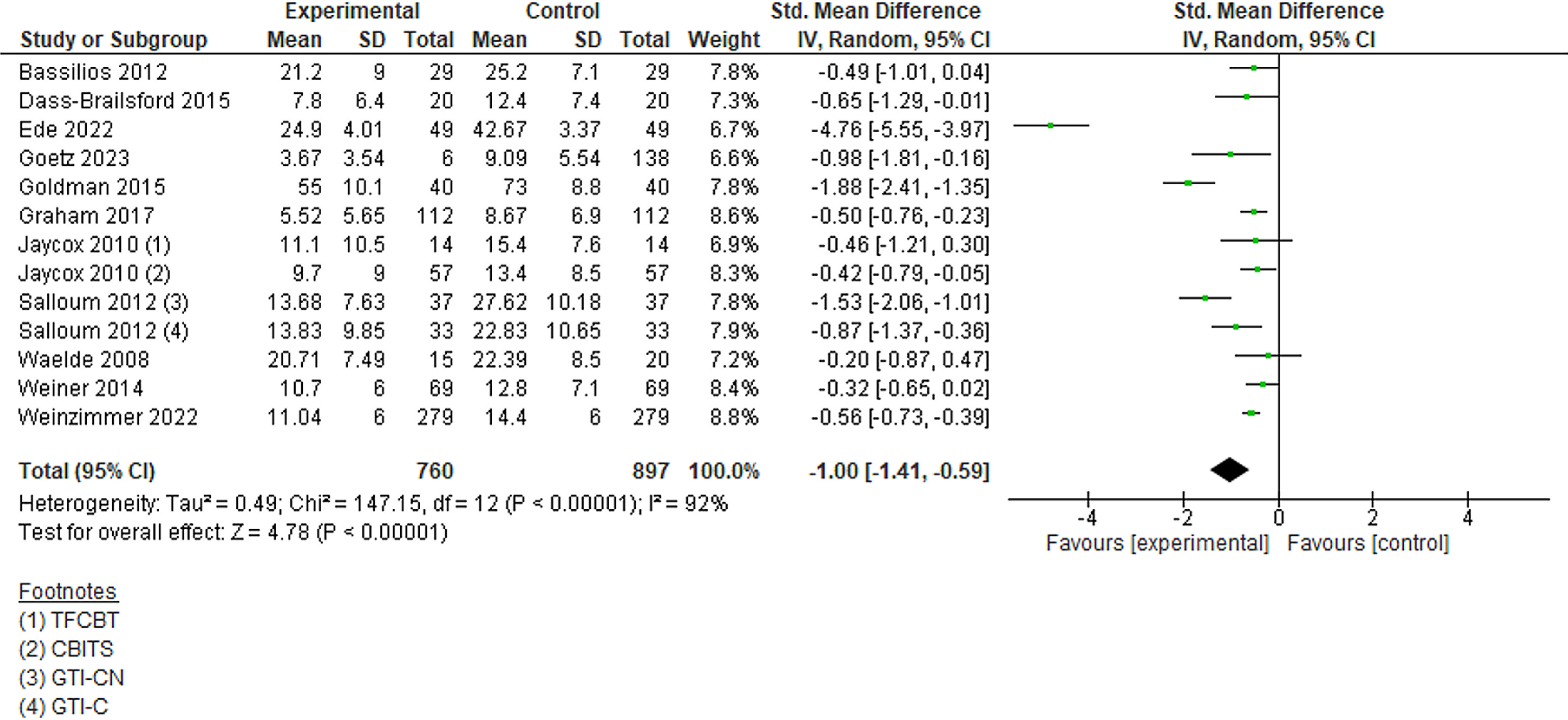
Depression symptoms (before-after studies).

Three intervention-control studies and nine before-after studies reported depression symptoms. Intervention-control studies showed a significant reduction in symptoms of depression (SMD −0.28, 95% CI (−0.53 to −0.03), three studies) compared with control. Analysis of before-after studies suggested a significant decrease in symptoms of depression after the intervention (SMD −1.00, 95% CI (−1.41 to −0.59), 11 studies) using various scales. Subgroup analysis of depression symptoms suggested a significant decrease of symptoms in HICs (intervention-control studies), adults and children both, and those affected by wildfires (intervention-control studies) and bushfires, floods and hurricanes (before-after studies) (figure 3, online supplemental figures 16–24 and table 1).

#### Anxiety

**Figure 4:**
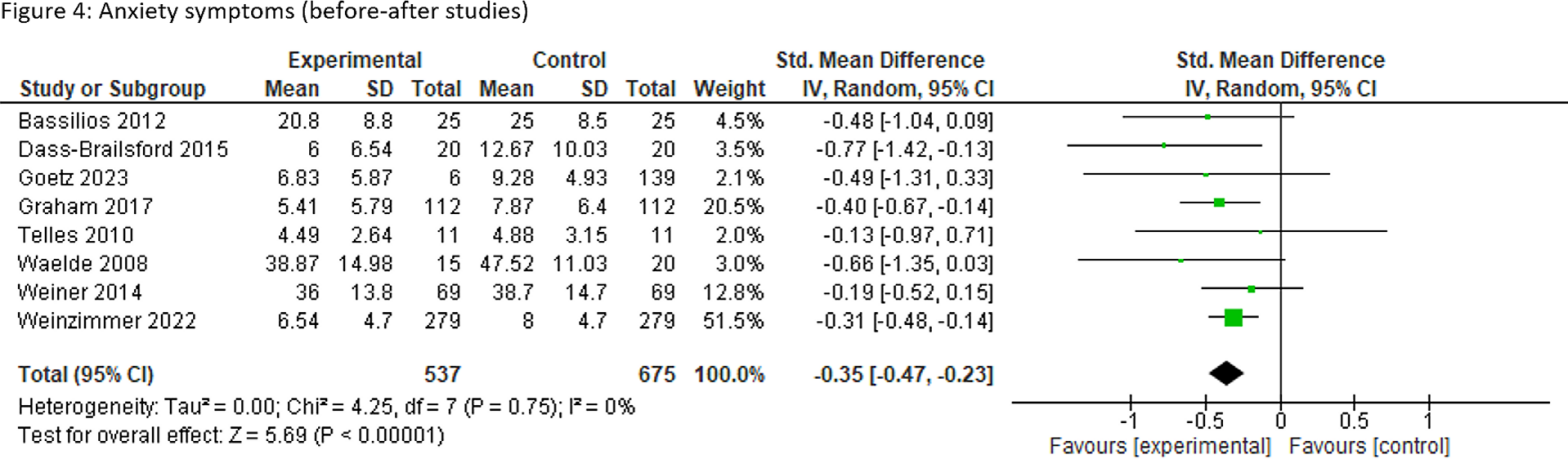
Anxiety symptoms (before-after studies).

Two intervention-control studies and six before-after studies reported anxiety symptoms. Anxiety symptoms were significantly improved in those who received mental health interventions compared with the control (SMD −0.38, 95% CI (−0.69 to −0.06), two studies). Before-after studies suggest a significant decrease in anxiety symptoms after the intervention (SMD −0.35, 95% CI (−0.47 to −0.23), eight studies) using various scales. Subgroup analysis showed a significant decrease in symptoms in HICs only and in adults (intervention-control studies) and both adults and children (before-after studies) and those affected by wildfires (intervention-control studies) and by hurricanes and tornadoes (before-after studies) (figure 4, online supplemental figures 25–33 and table 1).

#### Stress

Three intervention-control studies and two before-after studies reported stress symptoms. Stress symptoms were comparable in intervention-control studies after implementation of mental health interventions (SMD −0.18, 95% CI (−0.40 to 0.03), three studies). Before-after studies suggested a significant decrease in stress symptoms (SMD −0.82, 95% CI (−1.10 to −0.53), two studies). Subgroup analysis of stress symptoms suggested that symptoms decreased significantly in HICs and adults (before-after studies). There was a significant decrease in stress symptoms in people affected by bushfires (before-after studies) (online supplemental figures 34–43 and table 1).

#### General functioning impairment

Three intervention-control studies and two before-after studies reported general functioning impairment. In intervention-control studies, general functioning impairment was comparable in the intervention and control groups. In before-after studies, general functioning impairment decreased significantly after the intervention (SMD −1.22, 95% CI (−1.73 to −0.70), three studies). Subgroup analysis of general functioning impairment suggested a significant reduction in HICs as well as adults and children (before-after studies). There was a significant decrease in general functioning impairment in those affected by wildfires (intervention-control studies) and bushfires and hurricanes (before-after studies) (online supplemental figures 44–53 and table 1).

#### Well-being

Three intervention-control studies and two before-after studies reported well-being. Results from intervention-control studies suggested a significant improvement in well-being in individuals who received mental health interventions compared with controls (SMD 0.29, 95% CI (0.15 to 0.42), three studies). Before-after studies also suggested a significant improvement in well-being after the intervention (SMD 0.67, 95% CI (0.25 to 1.09), two studies). Subgroup analysis suggested significantly improved well-being in HICs (intervention-control studies) and both LMICs and HICs (before-after studies). Subgroup analysis by age showed a significant increase in well-being in adults but a comparable effect in children. Subgroup analysis based on EWE showed improved well-being in people affected by floods (intervention-control studies), typhoons and hurricanes (before-after studies) (online supplemental figures 54–63 and table 1).

## Discussion

This review identified interventions such as behavioural therapy, narrative therapy, yoga, psychoeducation, online resilience modules, etc, that were implemented in a variety of settings such as schools, camps and community centres, individually as well as in groups, to address mental health symptoms caused or aggravated by EWEs. Our overall estimates showed that individual as well as group interventions in both adult and paediatric populations alleviated a diverse range of symptoms, including PTSD, depression, anxiety and stress symptoms, to improve quality of life and overall functioning. The systematic review showed consistent impacts in HICs, while impacts in LMICs varied for different outcomes, but there were few studies from these settings. The evidence suggested consistent impacts on various mental health outcomes for EWE of wildfires, hurricanes and tornadoes compared with other EWE.

Several reviews have highlighted the psychological and emotional impacts of EWEs, pollution, increased heat, etc, and the complexity of the causal pathways that lead to climate-related mental health issues.^360^,^64^ These have resulted in several potentially effective mental health interventions being tested that involve schools, communities, trained healthcare workers and disaster volunteers using a range of therapies such as narrative counselling, cognitive and trauma-focused behavioural therapy, art therapy and group counselling. All interventions included in the review occurred after an EWE, some after basic needs and infrastructure had been settled and others in camps or temporary settings. Most focused on individual wellness and mental health improvement and involved frequent one-on-one sessions to process emotional responses to the disaster experienced. Some studies included trained psychologists who delivered the intervention, which might be difficult in many disaster-stricken areas, while other studies used the train-the-trainer method to reach larger numbers of affected people. Group interventions usually occurred in schools or community centres where shared experiences were the focus to enhance coping mechanisms, promote social cohesion and provide support to each other. Some school-based interventions were delivered by teachers trained in coping strategies and school counsellors.

While several studies have underscored the impact of long-term climate changes, such as increased temperature and heatwaves, leading to mood disorders, aggression, increased suicide risk, etc,^65^ we could not find any robust study on adaptation strategies and interventions to improve mood changes associated with heat.^4 66^ There is also limited evidence from LMICs, and more robust studies are needed to address challenges in resource-limited settings. Furthermore, while the mental health benefits of ecotherapy have been reported, no controlled trials of its effectiveness have been conducted even after the identification of psychoterratic syndromes of solastalgia, ecoanxiety, etc.^67 68^ There is a need for more robust studies from LMICs and the development of standardised training programmes for volunteers and healthcare and mental health professionals working in disaster settings. The programmes have to be cognisant of the cultural and ethical sensitivities of the specific settings.

Furthermore, several limitations and barriers to the implementation of interventions were identified. Long-term evaluation of the intervention was not done by any study included in this review and was especially difficult in settings where the intervention was carried out in temporary housing or shelters/camps. The outcome scales for mental health symptoms were very subjective and varied greatly across studies. The success of mental health improvement strategies depends on the willingness and capacity of individuals to engage in self-care; hence, lack of blinding and randomisation in most studies, coupled with some using self-report measures, could also potentially influence the reported impact of the interventions. The meta-analysis suggested high heterogeneity, confirming the variations among study settings, population, methods, interventions or other factors and hence a random-effects meta-analysis model was applied, which provides a simple parametric representation that allows effects to vary across studies and helps capture unobserved heterogeneity. The use of SMDs, because of varying scales of assessment, did not allow for assigning the units and absolute magnitude of the effect of an intervention. Also, population-specific or culture-sensitive interventions limit generalisability to broader populations of survivors. Evaluating mental health interventions in an acute post-weather event setting posed an ethical challenge to provide appropriate care to the comparison or control groups without having standardised treatment guidelines available for such situations.^23^ Furthermore, while train-the-trainer interventions, especially for mass events like floods, were considered, there was some loss of objectivity as some of the trainees themselves were survivors of the same weather event.^69^

The integration of mental health considerations into climate adaptation and EWE preparedness strategies is imperative to decrease the impact of the changing climate on mental health. This approach would emphasise the inextricable link between physical and mental well-being in the face of environmental adversity and the importance of taking a holistic approach to resilience-building, ensuring that psychological well-being is held in equal regard with physical health. Numerous guidelines, such as the Sendai Framework for Disaster Risk Reduction 2015–2030,^70 71^ Psychological First Aid and MHPSS^72^ developed by WHO and the Inter-Agency Standing Committee, exist to respond to mental health needs after disasters, but these are not specific to EWEs, highlighting the need for similar recommendations specifically in this context. These strategies should include risk awareness and assessment to anticipate the development of severe symptoms, as well as screening and identification processes to determine the necessity of psychosocial support, particularly for populations requiring climate adaptation efforts.^73^ Policies and strategies should also consider community resilience and coping through encouraging and building interpersonal and community resources within affected and vulnerable populations^74 75^ and also finding ways in which such support could be scaled up, which would vary by context.

There is also a need for longitudinal research endeavours to gauge the sustained impact of these mental health interventions, especially in frequently affected areas, and evaluate the development of tailored and culturally appropriate mental health response frameworks countering the effects of EWEs. The lack of quantitative studies investigating the link between EWEs and mental health within LMICs must be addressed in order to inform effective and sustainable interventions for affected communities.^15^ Additionally, there is a growing field of climate change-related mental health interventions using artificial intelligence for sustainability and long-term effectiveness which may be explored.^76^ Such innovative research may be able to combat the mental health impact of EWEs by co-designing AI resources and tools with affected communities and integrating these solutions into disaster management frameworks.^77^

The findings of this review will be of great value to policymakers, healthcare providers and researchers working in the field of climate change and mental health. By assessing effective interventions to address mental health issues resulting from EWEs, our research will contribute to the development of evidence-based policies and programmes to promote mental health and resilience in affected communities.

## Conclusion

As the frequency of extreme weather events increases, it is important to implement appropriate strategies to decrease their impact on affected populations. This review assesses the effect of several interventions on mental health symptoms and can provide valuable evidence to develop and implement effective, EWE-specific interventions and guidelines, but more evidence is needed, especially for long-term sustained effects and from low-resource settings.

## Supplementary material

10.1136/bmjgh-2025-020407 Supplementary file 1

10.1136/bmjgh-2025-020407 Supplementary file 2

## Data Availability

All data relevant to the study are included in the article or uploaded as supplementary information.
