# Supplementary material for: Systematic review and meta-analysis of interventions for mental health in extreme weather events
Source: BMJ Glob Health. 2026 May 28;11(5):e020407. doi: 10.1136/bmjgh-2025-020407 (PMC13223657; doi:10.1136/bmjgh-2025-020407)

**Systematic review and meta-analysis of interventions for mental health in extreme weather events**

Supplementary file

Search strategies …………………………………………………………………………………………………………………………………… 2

Study characteristics ……………...……………………………………………………………………………………………………………...7

Risk of bias assessment …………………………….…………………………………………………………………………………….….. 15

Meta analysis (forest plots)

Kaplan 2015 …………………..………………………………………………………………………………………………..……… 16

PTSD ……………………………………………………………………………………………………………………………….………. 17

Depression ……………………………………………………………………………………………………………….…………….. 22

Anxiety ………………………………………………………………………………………………………………………………..…. 26

Stress ………………………………………………………………………………………………………………………………..……. 30

General functioning impairment ……………………………………………………………………………………..……… 34

Wellbeing ……………………………………………………………………………………………………………………………….. 38

**Search Strategies**

**S1- Pubmed**

("Climate Change"[Mesh] OR "Natural Disasters"[Mesh] OR "Tsunamis"[Mesh] OR "Global Warming"[Mesh] OR "Cyclonic Storms"[Mesh] OR "Floods"[Mesh] OR "Tornadoes"[Mesh] OR "Sea Level Rise"[Mesh] OR "Wildfires"[Mesh] OR "Heat Stroke"[Mesh] OR "Air Pollution"[Mesh] OR "Droughts"[Mesh] OR "Tidal Waves"[Mesh] OR Urban Heat OR Urban Flood* OR Extreme Climate* OR Extreme Weather* OR “Indoor Air Pollution” OR “Household Air Pollution” OR “heat exposure” OR “extreme heat” OR “heat wave” OR “glaciers melt*” OR “smog” OR “avalanches” OR heat OR temperature OR “acid rain”)

**AND**

("Stress Disorders, Post-Traumatic"[Mesh] OR "Depression"[Mesh] OR "Anxiety"[Mesh] OR PTSD OR Anxious* OR Stress* OR “Substance-Related Disorders"[Mesh] OR "Schizophrenia Spectrum and Other Psychotic Disorders"[Mesh] OR "Panic Disorder"[Mesh] OR "Mental Health"[Mesh] OR "Mental Disorders"[Mesh] OR "Psychological Trauma"[Mesh] OR "Trauma and Stressor Related Disorders"[Mesh] OR "Suicide"[Mesh] OR "Resilience, Psychological"[Mesh] OR "Mood Disorders"[Mesh] OR "Disaster Victims"[Mesh] OR “Heat Stress Disorders”[Mesh] OR “Eco-Anxiety” OR “Ecological Grief” OR “EcoPsychology” OR Solastalgia OR “Psychoterratic Syndrome*” OR “Ecological Anxiety” OR “psychological health” OR “psychosocial health”)

**AND**

("Social Support"[Mesh] OR "Psychotherapy"[Mesh] OR "School Health Services"[Mesh] OR "Eye Movement Desensitization Reprocessing"[Mesh] OR "Adaptation, Psychological"[Mesh] OR "Community Health Services"[Mesh] OR "Disaster Planning"[Mesh] OR "Psychological First Aid"[Mesh] OR "Self-Help Groups"[Mesh] OR "Emergency Medical Services"[Mesh] OR "Psychosocial Intervention"[Mesh] OR "Implosive Therapy"[Mesh] OR "Narrative Therapy"[Mesh] OR "Behavior Therapy"[Mesh] OR "Relaxation Therapy"[Mesh] OR counsel* OR therapy* OR Eco-Therapy OR “Environmental Education”)

**S2- Cochrane**

ID Search Hits

#1 MeSH descriptor: [Climate Change] explode all trees 11

#2 MeSH descriptor: [Natural Disasters] explode all trees 62

#3 MeSH descriptor: [Tsunamis] explode all trees 2

#4 MeSH descriptor: [Cyclonic Storms] explode all trees 7

#5 MeSH descriptor: [Floods] explode all trees 5

#6 MeSH descriptor: [Tornadoes] explode all trees 5

#7 MeSH descriptor: [Wildfires] this term only 1

#8 sea level rise 315

#9 MeSH descriptor: [Heat Stroke] explode all trees 16

#10 MeSH descriptor: [Droughts] explode all trees 3

#11 MeSH descriptor: [Tidal Waves] explode all trees 2

#12 MeSH descriptor: [Extreme Weather] explode all trees 1

#13 MeSH descriptor: [Avalanches] explode all trees 5

#14 MeSH descriptor: [Smog] explode all trees 3

#15 MeSH descriptor: [Acid Rain] this term only 3

#16 extreme heat 220

#17 heat wave 187

#18 glaciers melting 0

#19 urban heat 67

#20 MeSH descriptor: [Stress Disorders, Traumatic] this term only 842

#21 MeSH descriptor: [Stress Disorders, Post-Traumatic] this term only 3149

#22 MeSH descriptor: [Mood Disorders] explode all trees 14184

#23 MeSH descriptor: [Anxiety Disorders] this term only 4571

#24 MeSH descriptor: [Substance-Related Disorders] explode all trees 16486

#25 MeSH descriptor: [Psychotic Disorders] explode all trees 3270

#26 MeSH descriptor: [Schizophrenia] this term only 8002

#27 MeSH descriptor: [Panic Disorder] this term only 985

#28 MeSH descriptor: [Psychological Trauma] this term only 111

#29 MeSH descriptor: [Suicide] explode all trees 1503

#30 MeSH descriptor: [Resilience, Psychological] explode all trees 303

#31 MeSH descriptor: [Disaster Victims] explode all trees 2

#32 heat stress disorder 91

#33 eco anxiety 40

#34 heat stress 1305

#35 ecological grief 3

#36 eco psychology 36

#37 solastalgia 0

#38 psychoterratic syndrome 0

#39 MeSH descriptor: [Social Support] explode all trees 3532

#40 MeSH descriptor: [Psychotherapy] explode all trees 27083

#41 MeSH descriptor: [School Mental Health Services] explode all trees 7

#42 MeSH descriptor: [Community Mental Health Services] explode all trees 753

#43 MeSH descriptor: [Disaster Planning] explode all trees 37

#44 MeSH descriptor: [Psychological First Aid] explode all trees 4

#45 MeSH descriptor: [Self-Help Groups] this term only 741

#46 Eye Movement Desensitization Reprocessing 432

#47 psychosocial intervention 11772

#48 eco therapy 294

#49 environmental education 2764

#50 community mobilisation 609

#51 community education 13239

#52 #1 or #2 or #3 or #4 or #5 or #6 or #7 or #8 or #9 #10 or #11 or #12 or #13 or #14 or #15 or #16 or #17 or #18 or #19 832

#53 #20 or #21 or #22 or #23 or #24 or #25 or #26 or #27 or #28 or #29 or #30 or #31 or #32 or #33 or #35 or #36 or #37 or #38 46803

#54 #39 or #40 or #41 or #42 or #43 or #44 or #45 or #46 or #47 or #48 or #49 or #50 or #51 54329

#55 #52 and #53 and #54

**S3- EBSCO CINAHL**

("Climate Change+" OR "Natural Disasters+" OR "Disaster Planning+" OR "Disasters+" OR "tsunami" OR "Greenhouse Effect" OR "global warming" OR "cyclone*" OR "cyclonic storm*" OR "flood*" OR "tornado*" OR "Sea Level Rise" OR "Wildfires" OR "Heat Stroke" OR "Air Pollution+" OR "Air Pollution, Indoor" OR "Air Pollutants, Environmental" OR "drought" OR "tidal waves" OR "Heat Exhaustion" OR "urban flood" OR "Extreme Weather" OR "extreme climate" OR "heat wave" OR "Smog" OR "avalanche" OR "acid rain") AND ("Stress Disorders, Post-Traumatic+" OR "Heat Stress Disorders+" OR "Depression+" OR "Seasonal Affective Disorder" OR "Anxiety+" OR "Anxiety Disorders+" OR "anxious" OR "Stress" OR "Substance Use Disorders+" OR "Alcohol-Related Disorders+" OR "Schizophrenia+" OR "Panic Disorder" OR "Mental Health" OR "Mental Disorders" OR "Psychological Trauma+" OR "Stress, Psychological+" OR "Suicide+" OR "Suicidal Ideation" OR "Adaptation, Psychological+" OR "psychological resilience" OR "Posttraumatic Growth, Psychological" OR "disaster victims" OR "ecological anxiety" OR "ecological grief" OR "eco psychology" OR "solastalgia" OR "psychoterratic syndrome" OR "Psychosocial Health+") AND ("Adaptation, Psychological+" OR "psychological resilience" OR "Posttraumatic Growth, Psychological" OR "eco psychology" OR "Psychotherapy+" OR "Cognitive Therapy+" OR "Support, Social+" OR "Support, Psychosocial+" OR "Social Work, Psychiatric" OR "Community Mental Health Services+" OR "Eye Movement Desensitization and Reprogramming" OR "Disaster Planning+" OR "Psychological First Aid" OR "Support Groups+" OR "Psychotherapy, Group+" OR "Psychosocial Intervention" OR "Internet-Based Intervention" OR "Rehabilitation, Psychosocial+" OR "implosive therapy" OR "narrative therapy" OR "Behavior Therapy+" OR "relaxation therapy" OR "eco therapy" OR "Mental Health Services+" OR "School Mental Health Services" OR “community mobiliz*” OR “community education”)

**S4- EMBASE**

1            exp climate change/ or greenhouse effect/          62378

2            natural disaster/             3905

3            disaster planning/ or tsunami/   15687

4            cyclone.mp. or hurricane/           6922

5            flooding/            8738

6            tornado/            507

7            sea level rise/ or air pollution/   69337

8            wildfire/             1830

9            drought/ or drought stress/        17661

10          climate change/ 50368

11          extreme weather/          384

12          depression/       435365

13          1 or 2 or 3 or 4 or 5 or 6 or 7 or 8 or 9 or 10 or 11             173448

14          posttraumatic stress disorder/ or stress disorders.mp.    75768

15          heat stress disorder.mp. or heat injury/ 3144

16          exp mood disorder/co, di, rh, th [Complication, Diagnosis, Rehabilitation, Therapy] 98342

17          psychological trauma.mp. or psychotrauma/       11666

18          suicide/ or suicide attempt/        90323

19          mental health/ or eco-anxiety/  180115

20          psychological adaptation.mp. or psychological adjustment/              2809

21          mental stress/ or solastalgia.mp. or distress syndrome/  147268

22          psychological resilience/ or adaptive behavior/  60623

23          ecological psychology/  21

24          exp psychotherapy/ or psychodynamic psychotherapy/ or interpersonal psychotherapy/ or short term psychotherapy/       277820

25          social support/  106662

26          mental health service/   61312

27          social support/ or psychological first aid/ or disaster planning/ or crisis intervention/         125834

28          web-based intervention/             1926

29          behavior therapy/ or psychotherapy/ or implosive therapy/              131185

30          relaxation therapy.mp. or relaxation training/     11963

31          narrative therapy/          462

32          school mental health service/     95

33          12 or 14 or 15 or 16 or 17 or 18 or 19 or 20 or 21              841080

34          22 or 23 or 24 or 25 or 26 or 27 or 28 or 29 or 30 or 31 or 32              496274

35          13 and 33 and 34            1191

36          limit 35 to human

**S5- Web of Science**

(Climate Change or Natural Disasters or Disaster Planning or Disasters or tsunami or Greenhouse Effect or global warming or cyclone* or cyclonic storm* or flood* or tornado* or Sea Level Rise or Wildfires or Heat Stroke or Air Pollution or indoor Air Pollution or drought or tidal waves or Heat Exhaustion or urban flood or Extreme Weather or extreme climate or heat wave or smog or avalanche or acid rain) and (Post traumatic Stress Disorders or Heat Stress Disorders or Depression or Seasonal Affective Disorder or Anxiety or Anxiety Disorders or anxious or Stress or Substance Use Disorders or Alcohol-Related Disorders or Schizophrenia or Panic Disorder or Mental Health or Mental Disorders or Psychological Trauma or Psychological Stress or Suicide or Suicidal Ideation or psychological resilience or Psychological Posttraumatic Growth or disaster victims or ecological anxiety or ecological grief or eco psychology or solastalgia or psychoterratic syndrome or Psychosocial Health) and (Psychological Adaptation or psychological resilience or Posttraumatic Growth or eco psychology or Psychotherapy or Cognitive Therapy or Social Support or psychosocial Support or Psychiatric Social Work or Community Mental Health Services or Eye Movement Desensitization and Reprogramming or Disaster Planning or Psychological First Aid or Support Groups or Group Psychotherapy or Psychosocial Intervention or Internet-Based Intervention or Psychosocial Rehabilitation or implosive therapy or narrative therapy or Behavior Therapy or relaxation therapy or eco therapy or Mental Health Services or School Mental Health Services or community mobilization or community education)

Table 1: Characteristics of included studies

| **S. No.** | **Study ID** | **Country** | **Study design** | **Extreme weather event** | **Intervention characteristics** | **Setting (school/clinic/camp)** | **Delivery (healthcare workers/disaster workers, etc)** | **Total duration of intervention** | **Duration of each session** | **No. of sessions** |
| --- | --- | --- | --- | --- | --- | --- | --- | --- | --- | --- |
| 1 | Bassilios 2012^19^ | Australia | Before-after | bushfire | psychoeducation, CBT, interpersonal therapy, narrative therapy | clinic | general practitioners and allied health workers |  | 46-60mins | average 6.5 |
| 2 | Chemtob 2002^20^ | Hawaii | Controlled Community Field Study | hurricane | 4 sessions with different themes like loss, etc | school | school based counselor | 4 weeks |  | 1/week |
| 3 | Crombach 2018^21^ | Burundi, Eastern Africa | Quasi-experimental | flood | Narrative exposure therapy | camp | final year undergraduate psychology students from the University Lumière of Bujumbura | 6 weeks | 1.5-2.5 hours | 1/week |
| 4 | Ede 2022^22^ | Nigeria | Waitlist control | flood | Rational emotive behavioural therapy |  | therapists | 12 weeks | 50 mins | 20 |
| 5 | Gibson 2021^23^ | Tuvalu | quasi experimental control design | cyclone | Skills fOr Life Adjustment and Resilience (SOLAR) targeted distress and impairment following disaster and trauma. |  | trained, non-specialist or lay ‘Coaches’. | 5 days |  | 5 |
| 6 | Gilmore 2021, Ruggiero 2015^24,25^ | USA | RCT | tornado | Bounce Back Now (BBN) included four-session modules on PTSD symptoms depressive symptoms cigarette use and alcohol use |  |  |  |  | 4 |
| 7 | Goldman 2015^26^ | USA | Before-after | hurricane | intervention groups targeted depression or disruptive behavior |  | clinician psychotherapy researchers, the IPC, who trained STEP clinicians |  |  | 8 to 12 |
| 8 | Graham 2017^27^ | USA | Before-after | hurricane | modified versions of CBT models including trauma focused CBT (TF-CBT) and Cognitive Behavioral Interventions in Schools (CBITS). | school | psychologists |  | 55 mins | mean 26.4 sessions per year |
| 9 | Hamblen 2009^28^ | USA | Quasi experimental time series design | hurricane | CBT-PD | clinic | therapists |  |  | 10 |
| 10 | Hechanova 2015^29^ | Phillipinese | Before-after | typhoon | brief mindfulness training, positive coping strategies, psychoeducation session. | survivors were transported and lodged in a location not directly affected by the typhoon. |  |  |  |  |
| 11 | Jaycox 2010^30^ | USA | Before-after | hurricane | Cognitive-Behavioral Intervention for Trauma in Schools (CBITS) and Trauma-Focused Cognitive Behavioral Therapy(TF-CBT) | school | therapists |  |  | 11 to 13 |
| 12 | Jones 2009^31^ | USA | Before-after | hurricane | specialized crisis counseling services (SCCS) | crisis centers | counselors |  |  | median 3 |
| 13 | Jordans 2021^32^ | Nepal | RCT | annual floods | evidence-based techniques: (a) problem- solving; (b) stress management through deep breathing; (c) behavioral activation; and (d) promoting social support. |  |  | 5 weeks | 2.5 hours | 5 |
| 14 | Kaplan 2015^33^ | Canada | RCT | flood | B-Complex with MetafolinTM (NPN 80021762) in large transparent gelatin capsules. |  |  |  |  |  |
| 15 | Leitch 2009^34^ | USA | Quasi experimental | hurricane | interventions that focus on self-regulation and on working with associated emotions and cognitions. | food warehouses, walk-in clinics, and offices etc | clinicians |  | 40 to 60 mins | 1 to 2 |
| 16 | Mahaffey 2020^35^ | USA | RCT | hurricane | Psychological techniques such as motivational interviewing, health behavior goal setting, and relaxation training |  | trained mental health professionals (clinical psychologist or social worker) | 1 session workshop | 4 hrs | 1 |
| 17 | Mathew 2021^54^ | India | Before-after | flood | slow breathing technique like Nadishuddhi pranayama and Brahmari pranayama |  |  | 15 days | 30 mins | 15 |
| 18 | Oba 2010^36^ | Thailand | Before-after | flood and landslide | Potential enhancement program (PEP)- involved health education and dissemination (physical health, stress management etc) | village | health volunteers | 19 weeks |  | 1/week |
| 19 | O'Donnell 2020^37^ | Australia | Before-after | bushfires | SOLAR, constitutes a 5-session psychosocial intervention | any easily accessible location in the community | volunteers, professionals, or paraprofessionals working in health or disaster response | 5 weeks | 50 mins (1st session 80 mins) | 5 |
| 20 | Salloum 2012^38^ | USA | Before-after | hurricane | techniques from CBT and narrative therapy | school | clinicians | 10 weeks | 50-60 mins | 12 |
| 21 | Weiner 2014^39^ | USA | Before-after | hurricane | Guzman and Nicassio’s cognitive/behavioral framework of illness schema in dialysis patients | hospital | social workers |  | 10 to 29 mins | 5 |
| 22 | Sirey 2020^41^ | USA | Before-after | hurricane | Reward-based psychotherapy intervention for late-life depression | local senior center/nutrition sites | licensed social workers or mental health counselors | 6 weeks | 45 mins | 6 |
| 23 | Steinmetz 2012^40^ | USA | RCT | hurricane | interactive modules on seeking professional help, relaxation, social support, unhelpful ways of coping, self-talk, and trauma triggers and memories. | web based |  | 30 days |  | 6 |
| 24 | Telles 2010^42^ | India | RCT | flood | yoga class included loosening exercises, physical postures and breathing techniques and relaxation. | camp |  | 7 days | 1 hour | 7 |
| 25 | Waelde 2008^43^ | USA | Before-after | hurricane | meditation workshop. |  |  | 8 weeks | 30 mins | 6 days/week |
| 26 | Dass-Brailsford 2015^44^ | USA | Before-after | hurricane | Walk-in counseling services (WICS) modeled after the Minnesota Walk-in Counseling Center | local church | Licensed social workers, psychologists, and counselors | as many as needed by participant |  | >=8 |
| 27 | Powell 2016^45^ | USA | Quasi experimental waitlist control | tornado | build healthy coping skills & target specific emotions related to disaster exposure | school | master’s level mental health professionals (i.e., counselors, social workers, psychologists) with previous experience in group work |  | 1 hour | 8 sessions |
| 28 | Powell 2022^46^ | USA and Puerto Rico | Before-after | hurricane | Resilience and Coping for the Healthcare Community and its expanded version |  | 2 facilitators at least one of whom is a licensed mental health professional |  | 3 hours followed by 1 hour | 2 |
| 29 | Weinzimmer 2022, Goetz 2023 ^4847,^ | USA | Before-after | hurricane | Unified Protocol (UP) for Transdiagnostic Treatment of Emotional Disorders | community | Master’s level therapists |  | 30 mins | Range 2-21 (mean 5.22) |
| 30 | Frenette 2023, Belleville 2023 ^49,50^ | Canada | RCT | wildfire | internet based cognitive behavioural therapy (ICBT) | online | trained therapists | 6 months |  | 12 |
| 31 | Apuke 2023 ^51^ | Nigeria | Quasi experimental | flood | art, music and poetry therapy | Whatsapp-based | certified therapists | 3 weeks | 1 hour | 8 |
| 32 | Cain 2010 ^52^ | USA | Before-after | hurricane | Weathering The Storm: Psychological First Aid (WTS PFA) | School or educational buildings located within trailer parks | licensed social workers | 6 weeks | 1 hour |  |
| 33 | Cowlishaw 2023^53^ | Australia | RCT | wildfire and drought | SOLAR | Online or in-person if feasible | volunteers with prior counselling experience or employees of community service organisations | 5 weeks |  | 5 |

Figure 1: Risk of bias assessment using ROB


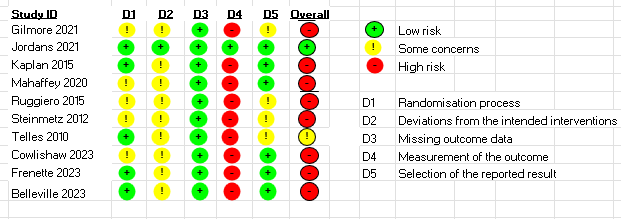


Figure 2: Risk of bias assessment using ROBINS


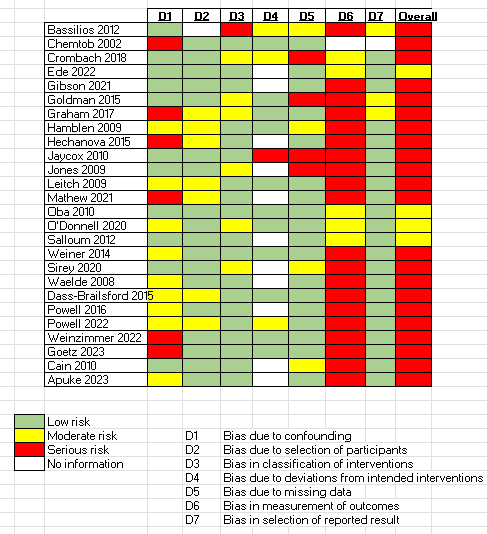


Figure 3: PTSD symptoms in Kaplan


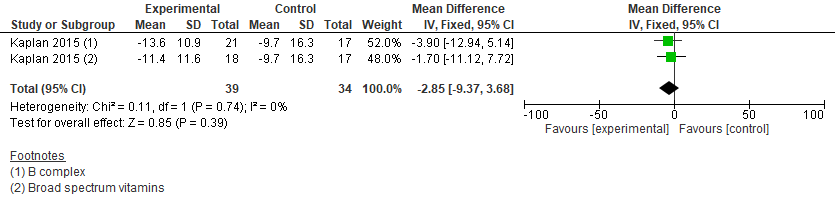


Figure 4: Depression symptoms in Kaplan


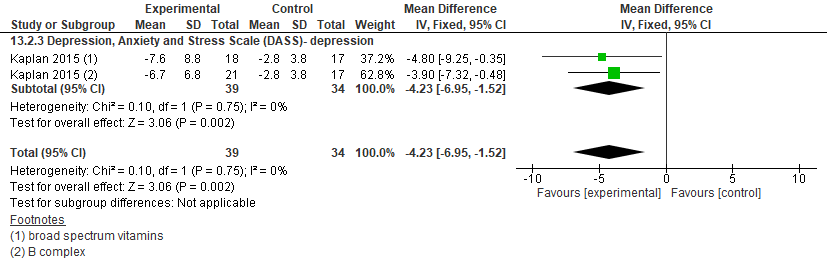


Figure 5: Anxiety symptoms in Kaplan


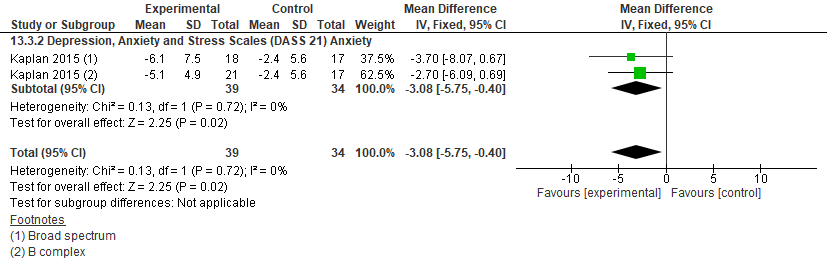


Figure 6: Stress in Kaplan 2015


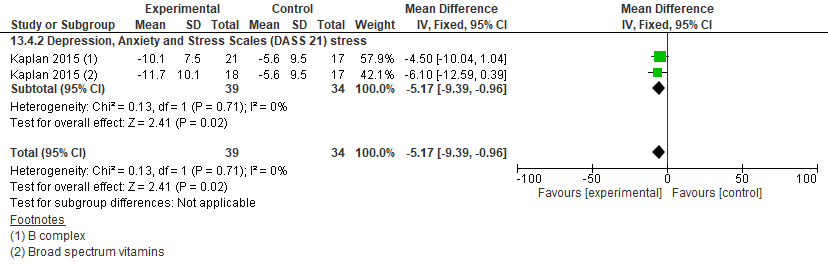


Figure 7: PTSD symptoms in intervention-control studies (subgroups by scales)


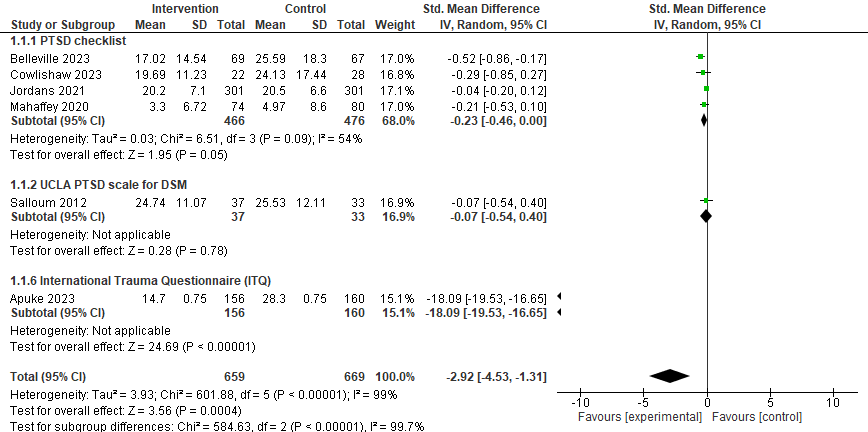


Figure 8: PTSD symptoms in intervention-control (subgroups by SES)


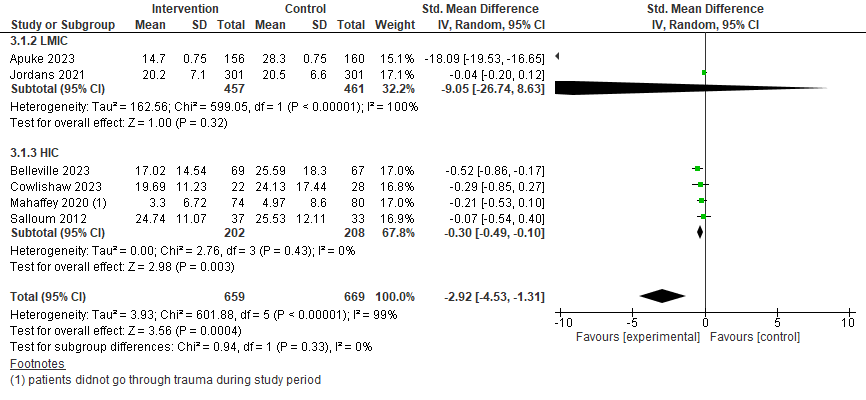


Figure 9: PTSD symptoms in intervention-control studies (subgroups by age)


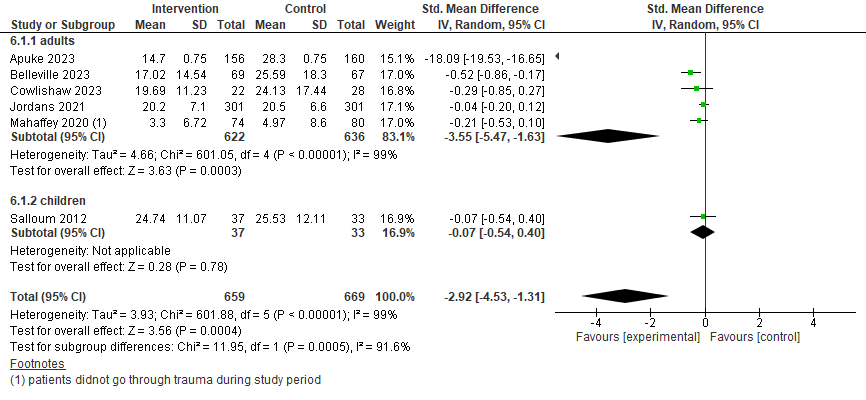


Figure 10: PTSD symptoms in intervention-control studies (subgroups by extreme weather events)


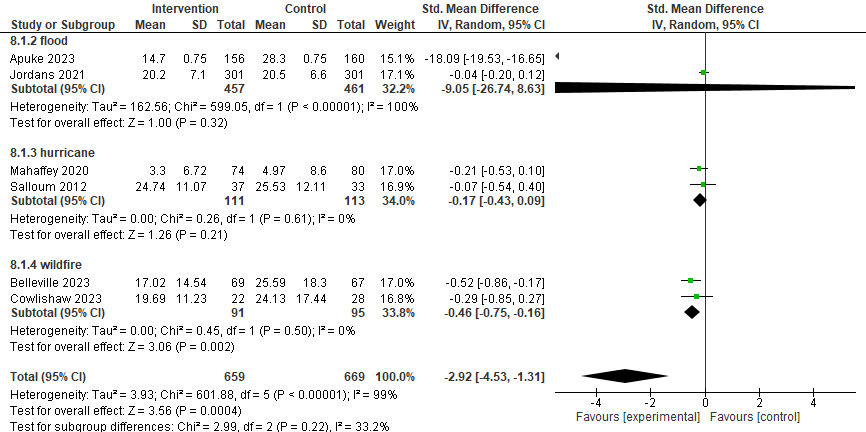


Figure 11: PTSD symptoms in before-after studies


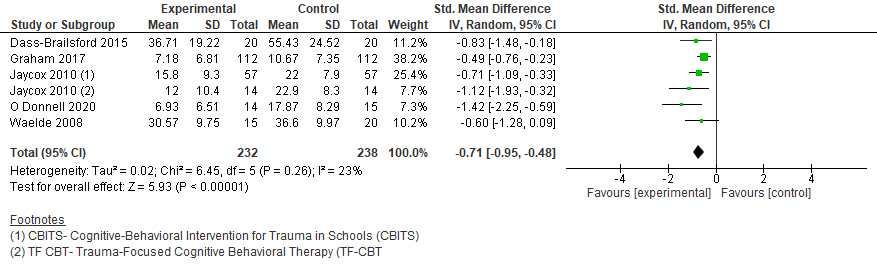


Figure 12: PTSD symptoms in before-after studies (subgroups by scales)

**
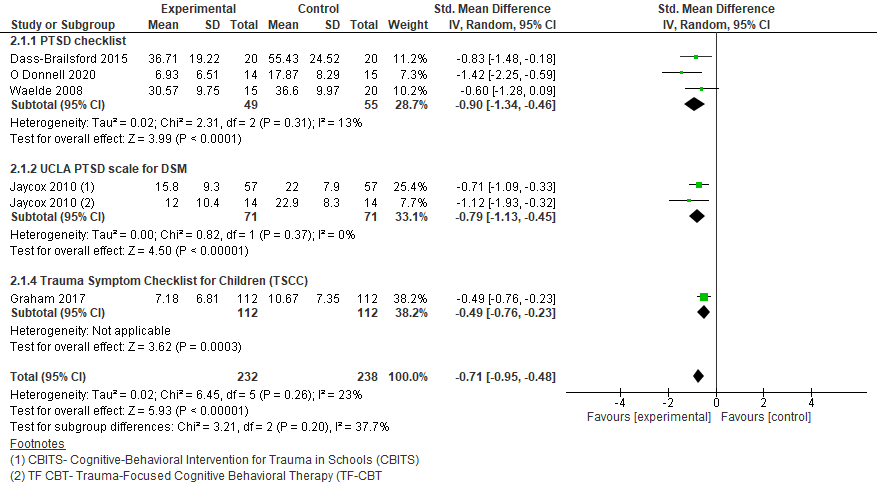
**

Figure 13: PTSD symptoms in before-after studies (subgroups by SES)


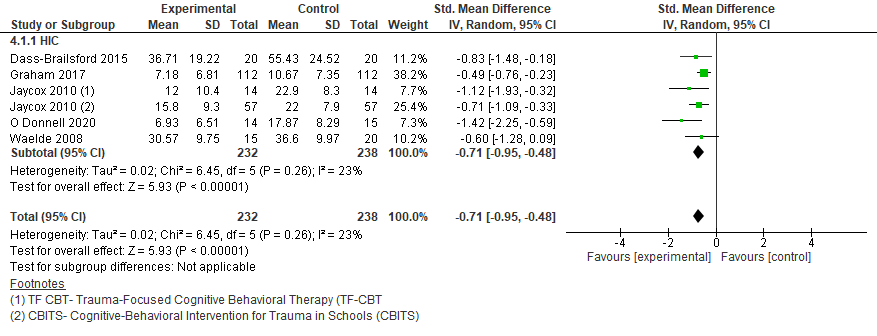


Figure 14: PTSD symptoms in before-after studies (subgroups by age)


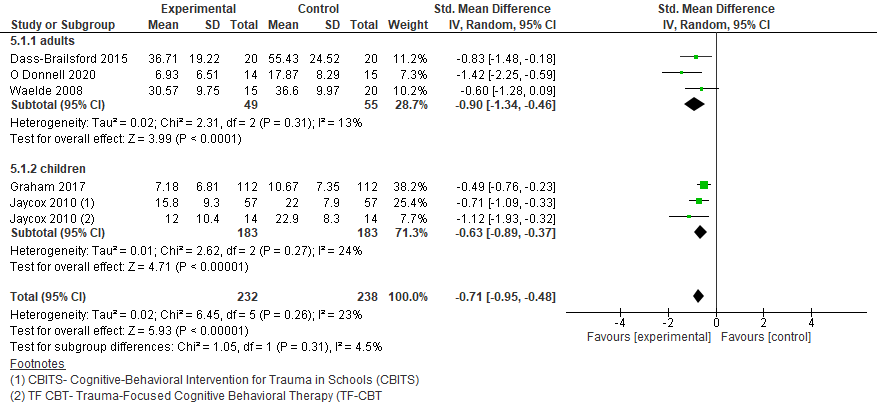


Figure 15: PTSD symptoms in before-after studies (subgroups by extreme weather event)


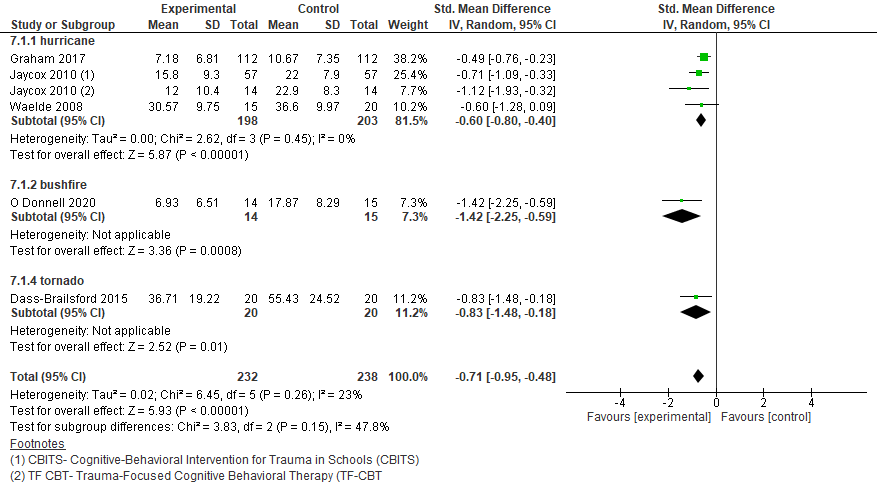


Figure 16: Depression symptoms in intervention-control studies


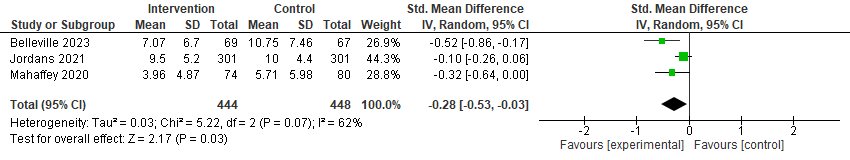


Figure 17: Depression symptoms in intervention-control studies (subgroups by scales)


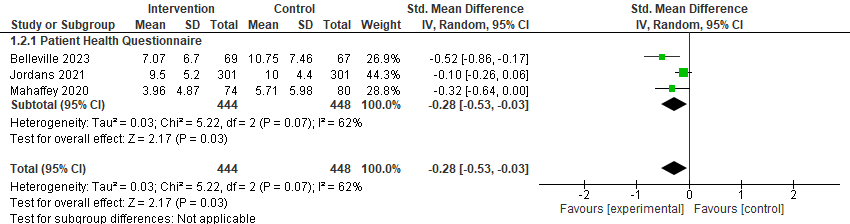


Figure 18: Depression symptoms in intervention-control studies (subgroups by SES)


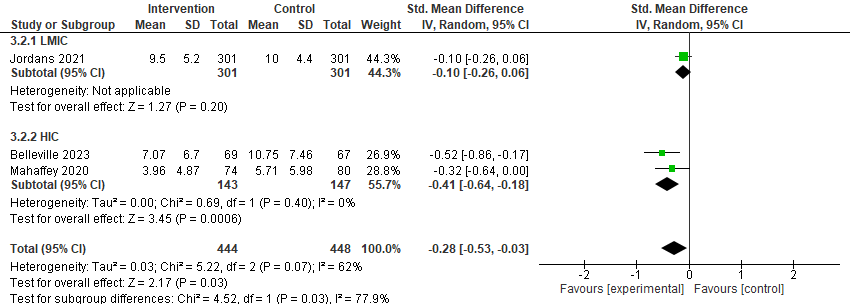


Figure 19: Depression symptoms in intervention-control studies (subgroups by age)


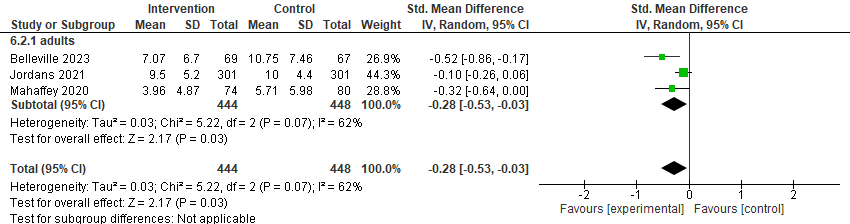


Figure 20: Depression symptoms in intervention-control studies (subgroups by extreme weather events)


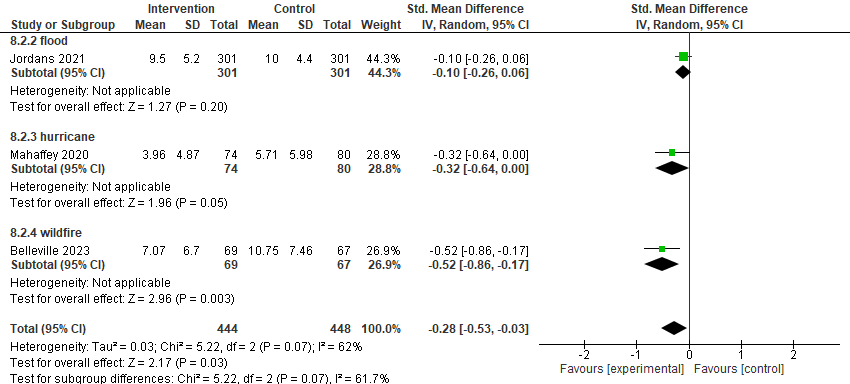


Figure 21: Depression symptoms in before-after studies (subgroups by scales)


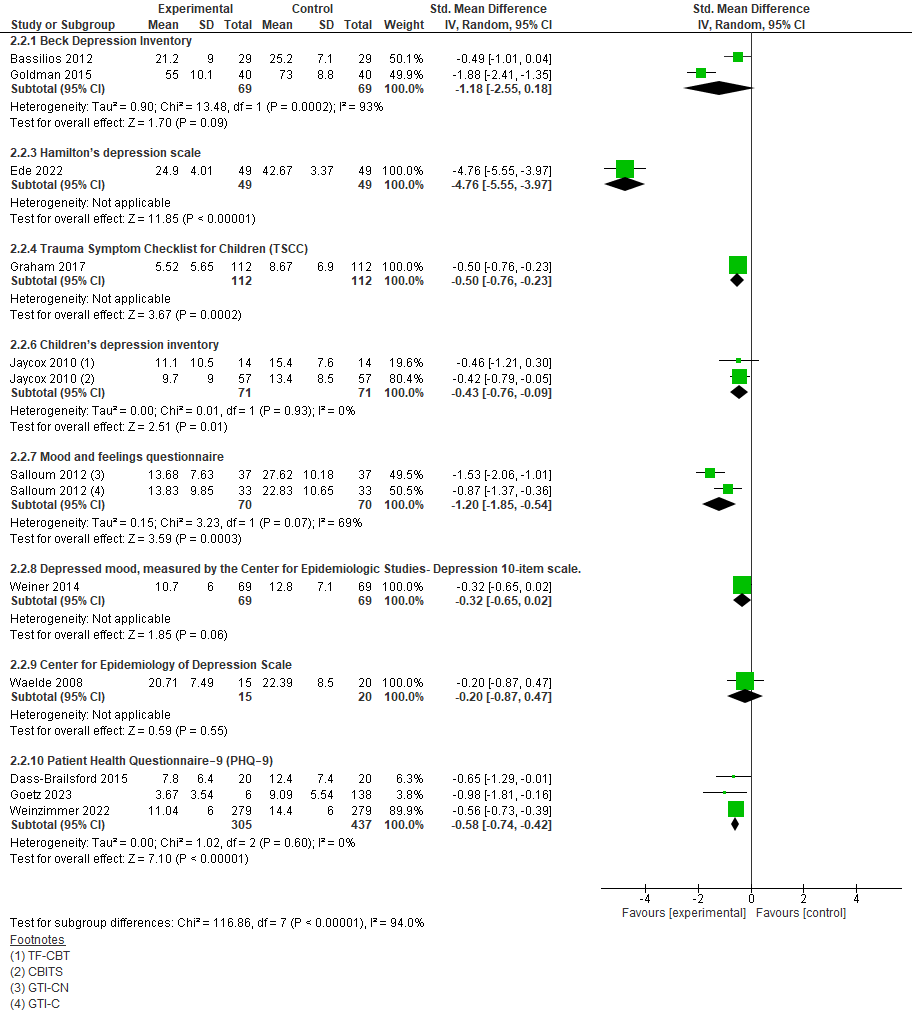


Figure 22: Depression symptoms in before-after studies (subgroups by SES)


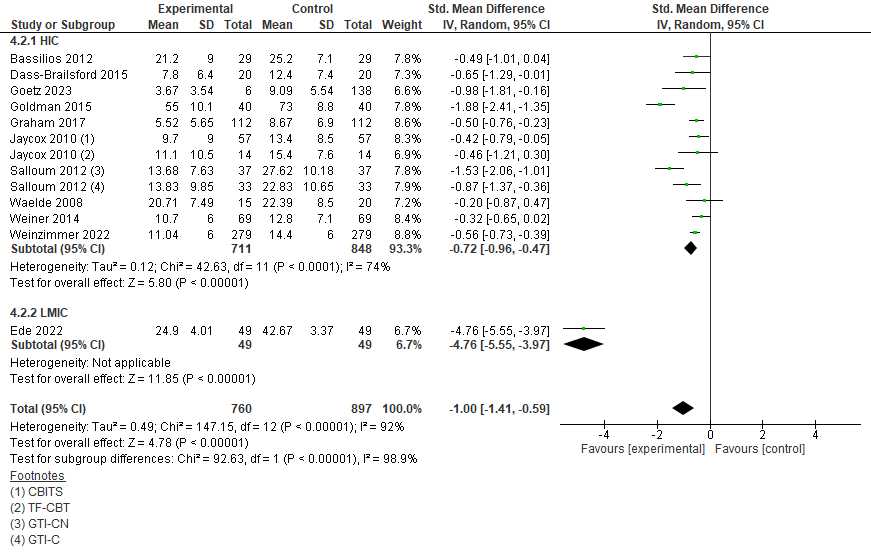


Figure 23: Depression symptoms in before-after studies (subgroups by age)


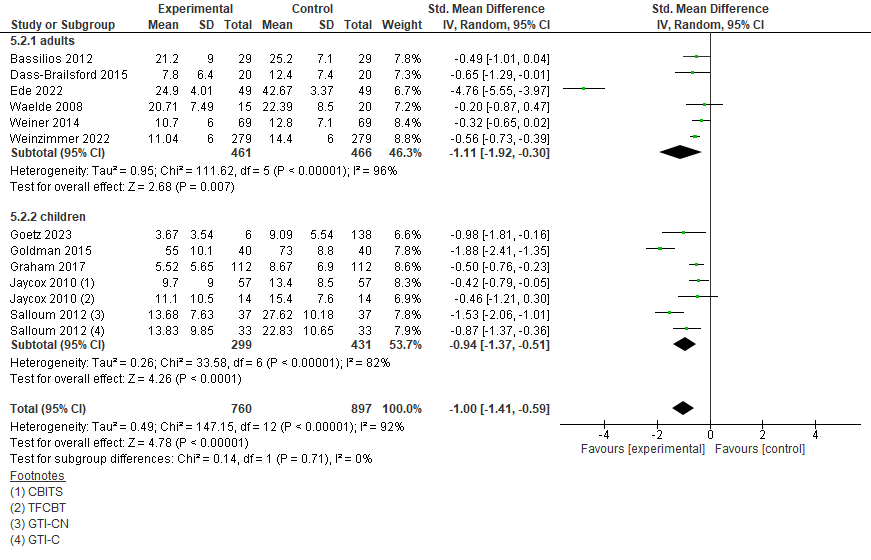


Figure 24: Depression symptoms in before-after studies (subgroups by extreme weather events)


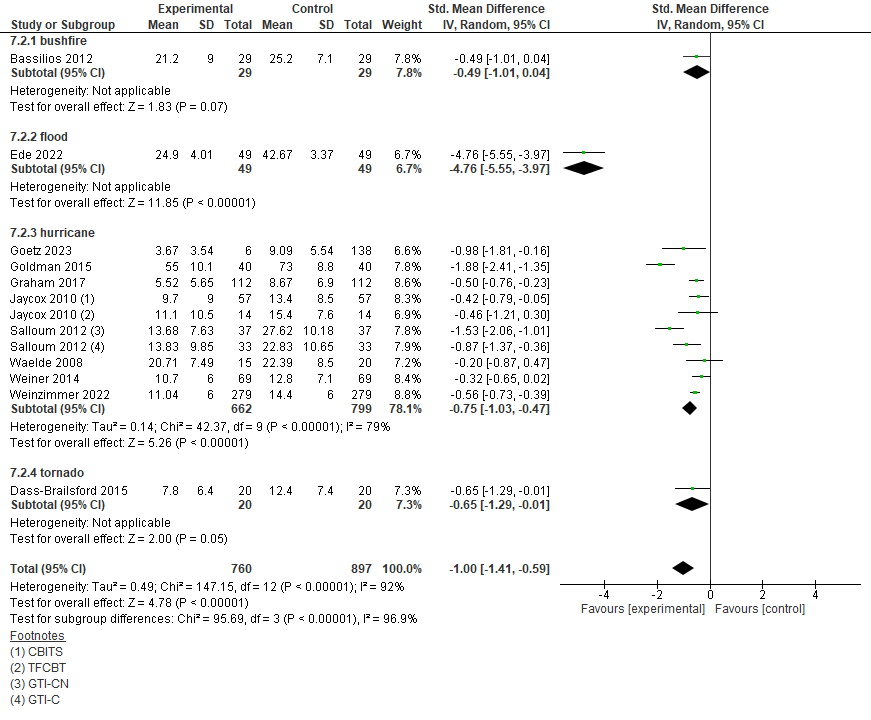


Figure 25: Anxiety symptoms in intervention-control studies


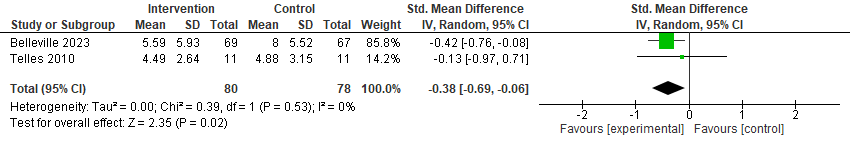


Figure 26: Anxiety in intervention-control studies (subgroups by scales)

Figure 27: Anxiety symptoms in intervention-control studies (subgroups by SES)


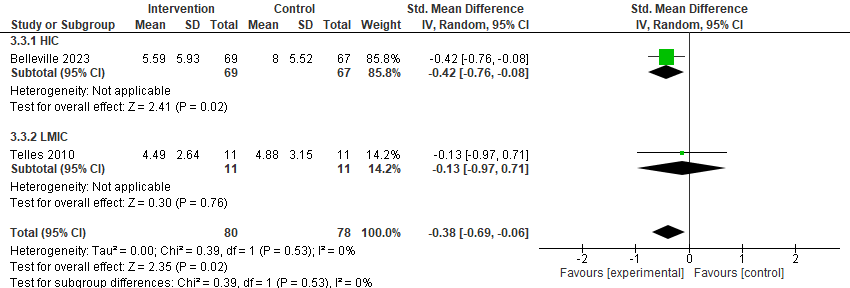


Figure 28: Anxiety symptoms in intervention-control studies (subgroups by age)


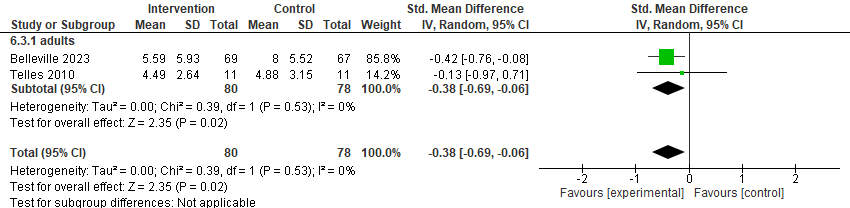


Figure 29: Anxiety symptoms in intervention-control studies (subgroups by extreme weather events)


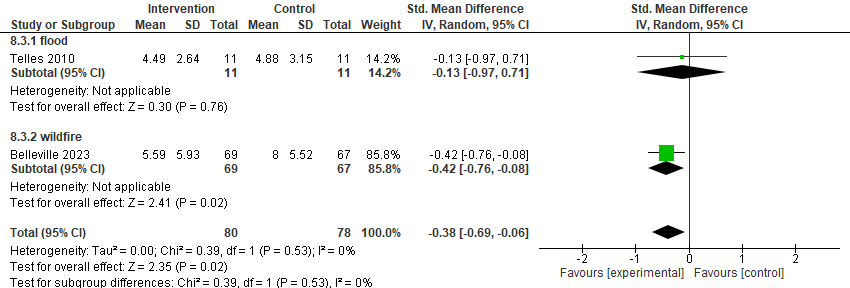


Figure 30: Anxiety symptoms in before-after studies (subgroups by scales)


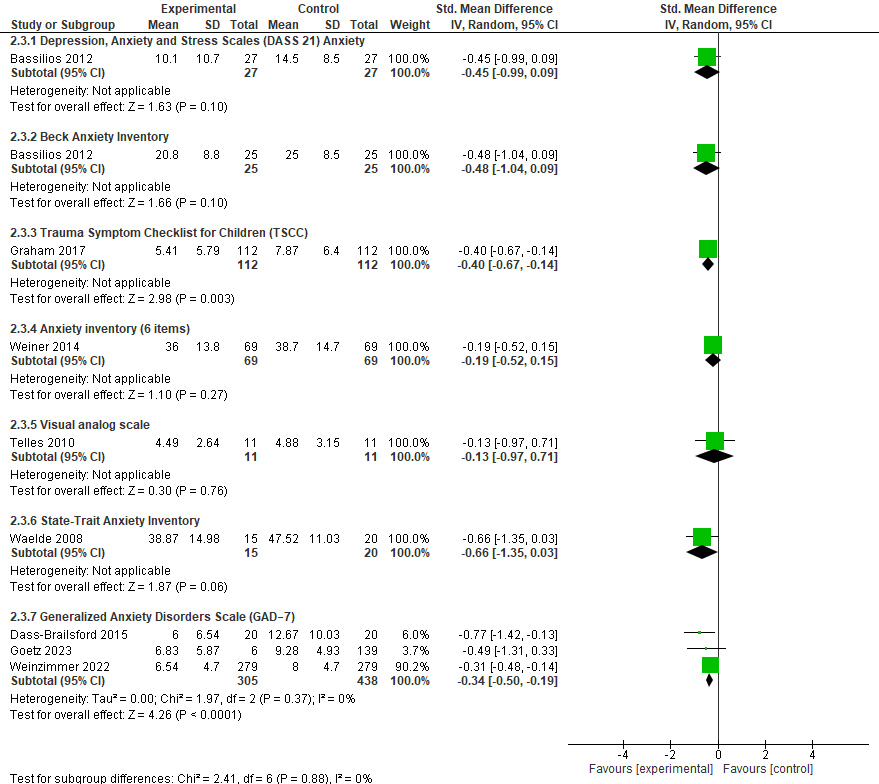


Figure 31: Anxiety symptoms in before-after studies (subgroups by SES)


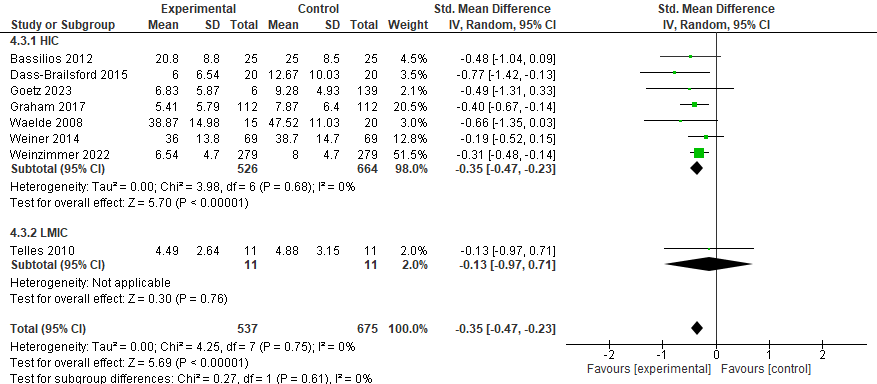


Figure 32: Anxiety symptoms in before-after studies (subgroups by age)


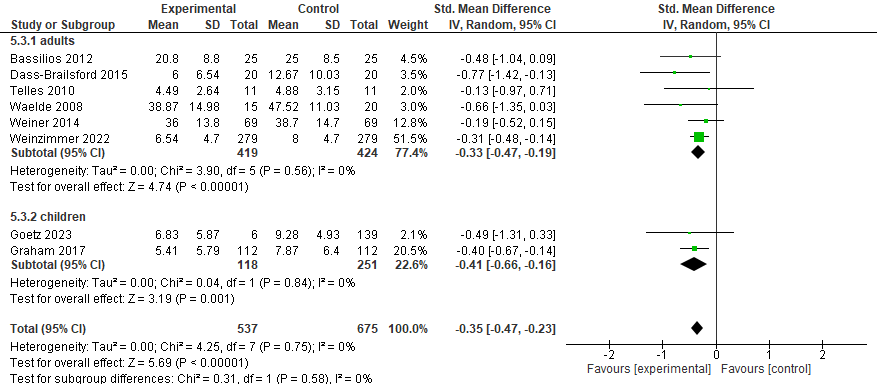


Figure 33: Anxiety symptoms in before-after studies (subgroups by extreme weather events)


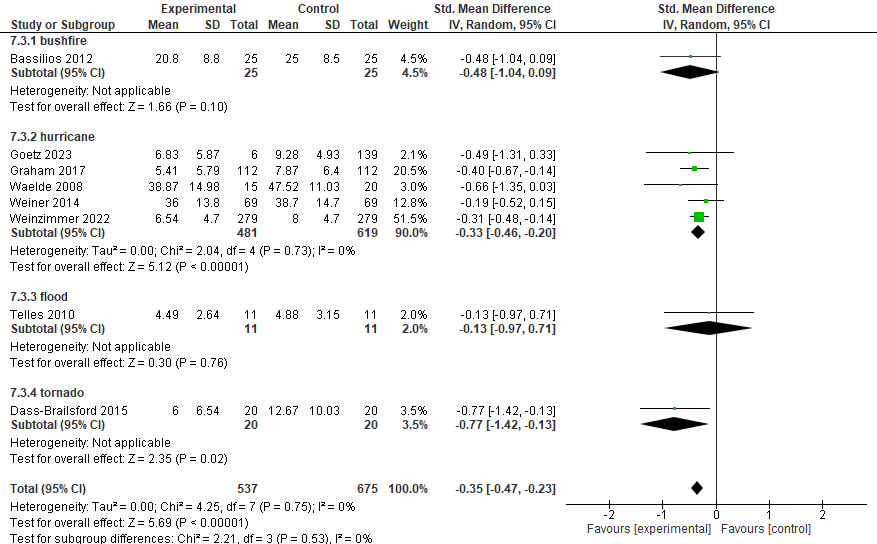


Figure 34: Stress symptoms in intervention-control studies


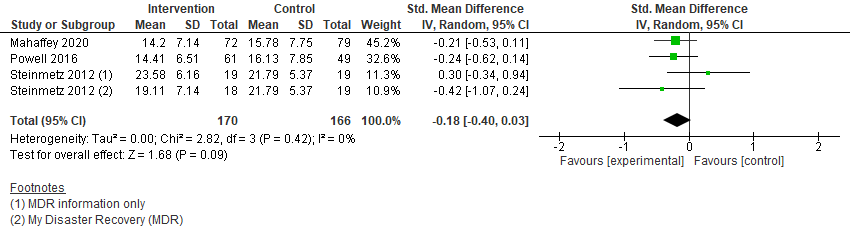


Figure 35: Stress symptoms in intervention-control studies (subgroups by scales)


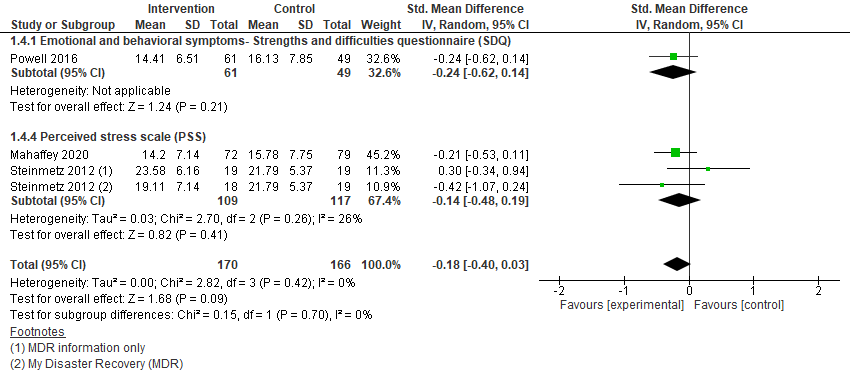


Figure 36: Stress symptoms in intervention-control studies (subgroups by SES)


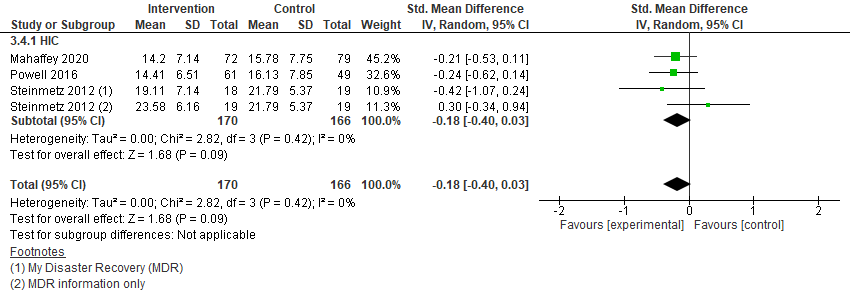


Figure 37: Stress symptoms in intervention-control studies (subgroups by age)


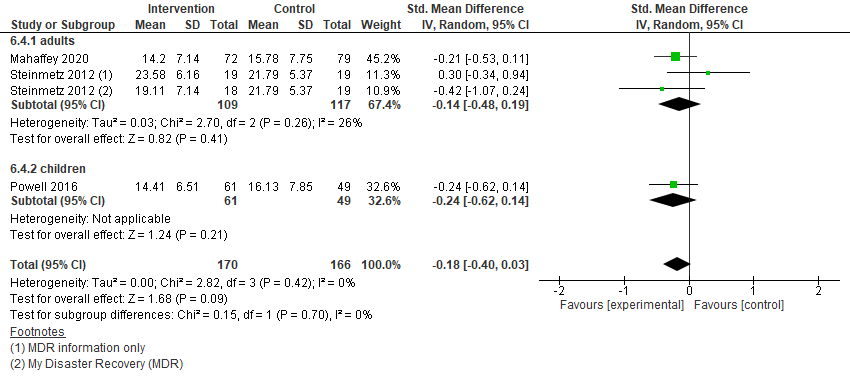


Figure 38: Stress symptoms in intervention-control studies (subgroups by extreme weather events)


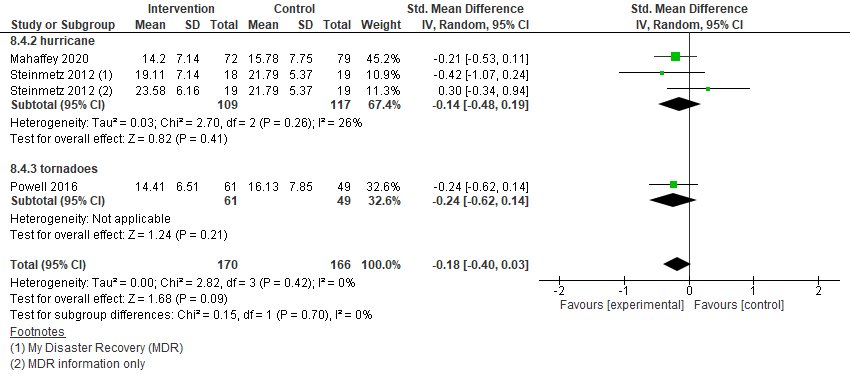


Figure 39: Stress symptoms in before-after studies


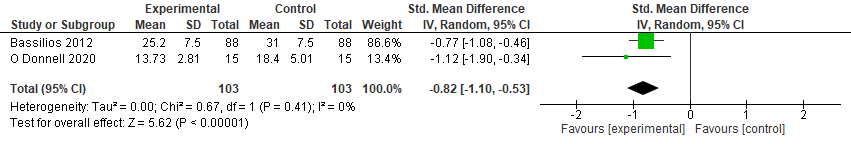


Figure 40: Stress symptoms in before-after studies (subgroups by scales)


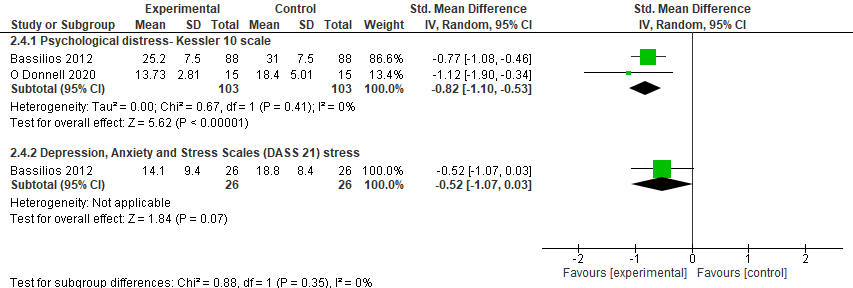


Figure 41: Stress symptoms in before-after studies (subgroups by SES)


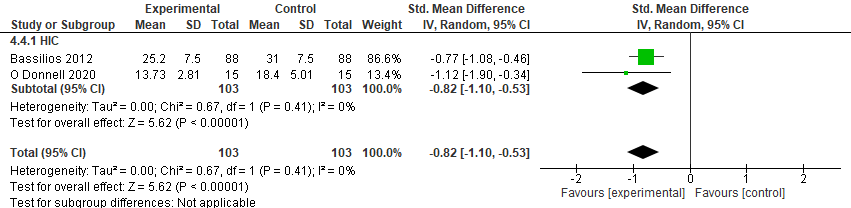


Figure 42: Stress symptoms in before-after studies (subgroups by age)


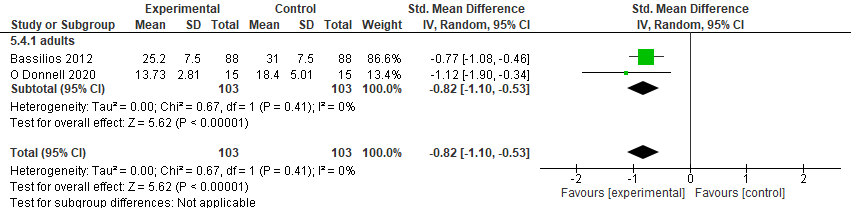


Figure 43: Stress symptoms in before-after studies (subgroups by extreme weather events)


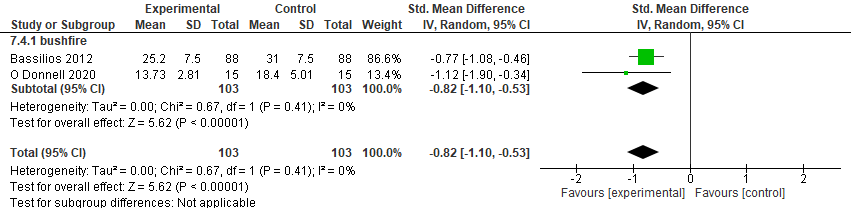


Figure 44: General functioning impairment in intervention-control studies


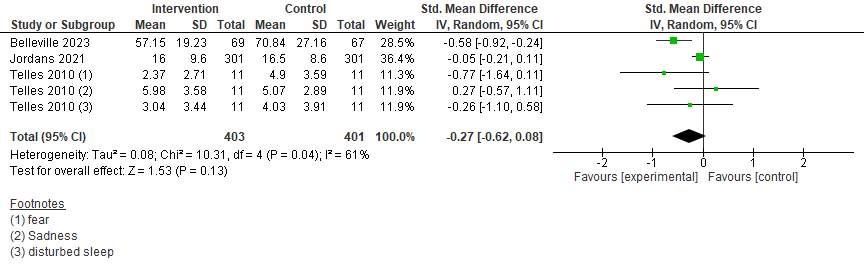


Figure 45: General functioning impairment in intervention-control studies (subgroups by scales)

**
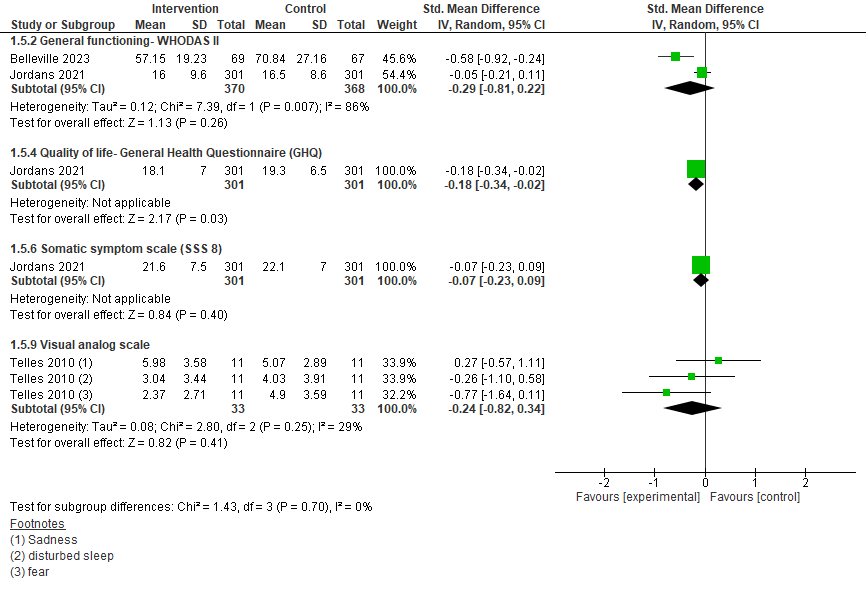
**

Figure 46: General functioning impairment in intervention-control studies (subgroups by SES)


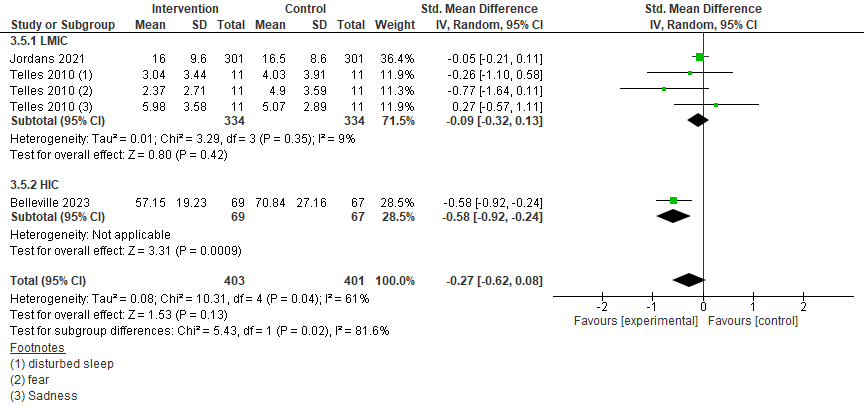


Figure 47: General functioning impairment in intervention-control studies (subgroups by age)


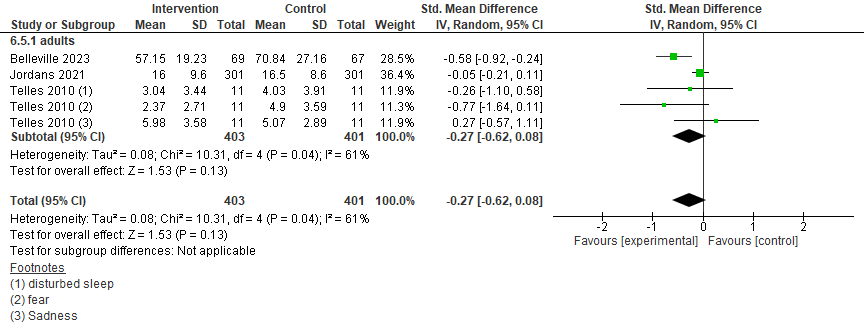


Figure 48: General functioning impairment in intervention-control studies (subgroups by extreme weather events)


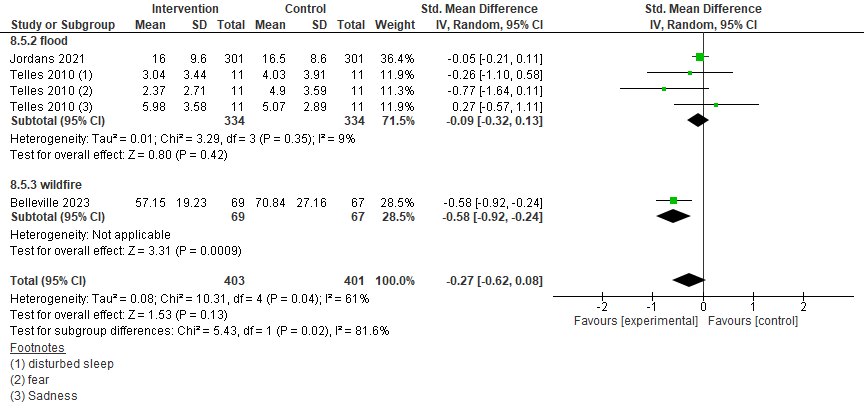


Figure 49: General functioning impairment in before-after studies


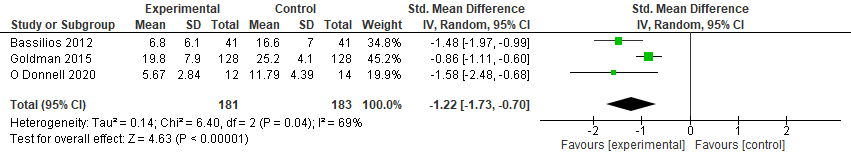


Figure 50: General functioning impairment in before-after studies (subgroups by scales)


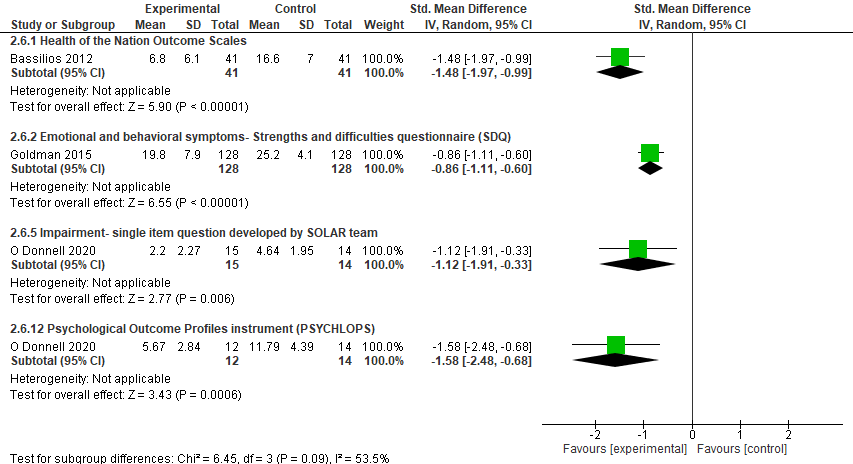


Figure 51: General functioning impairment in before-after studies (subgroups by SES)


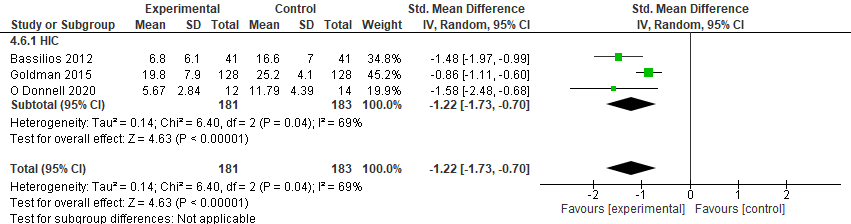


Figure 52: General functioning impairment in before-after studies (subgroups by age)


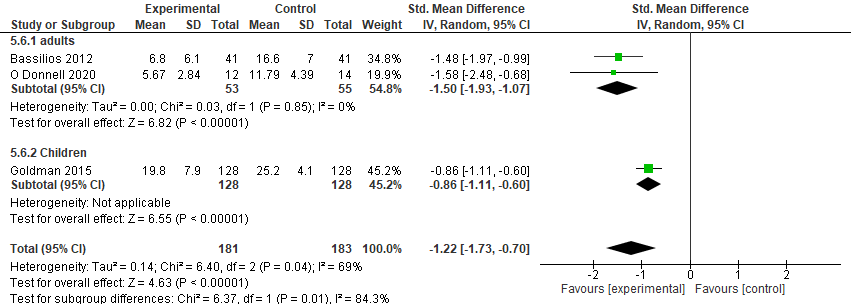


Figure 53: General functioning impairment in before-after studies (subgroups by extreme weather events)


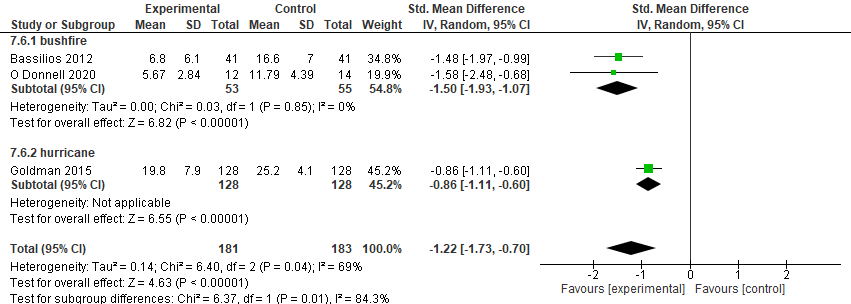


Figure 54: Wellbeing in intervention-control studies


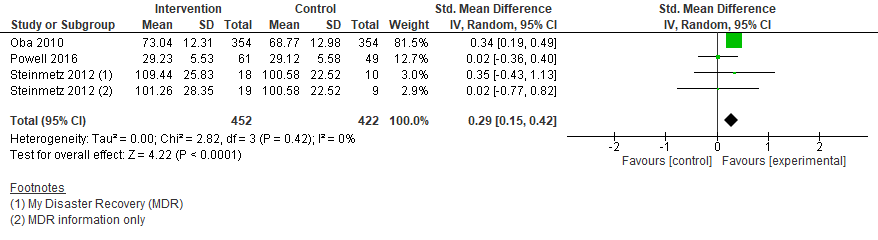


Figure 55: Wellbeing in intervention-control studies (subgroups by scales)


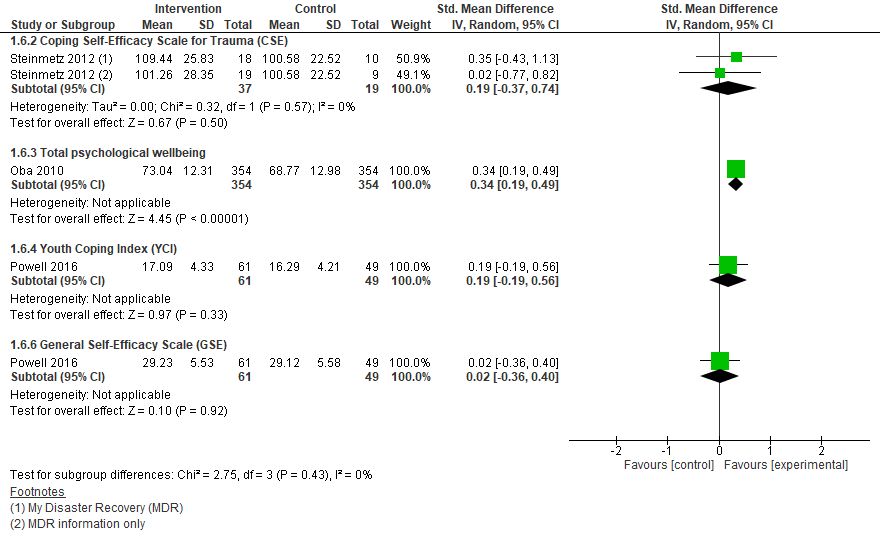


Figure 56: Wellbeing in intervention-control studies (subgroups by SES)


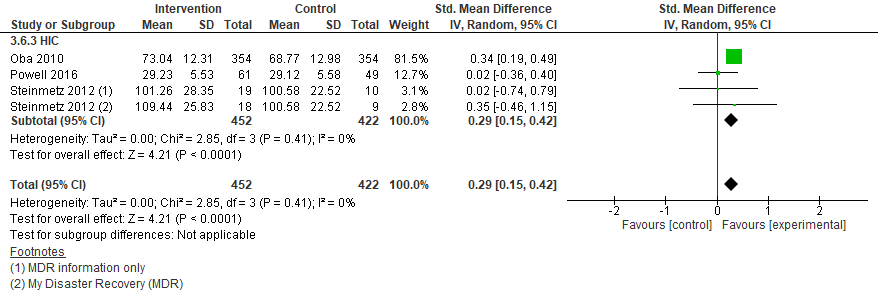
Figure 57: Wellbeing in intervention-control studies (subgroups by age)


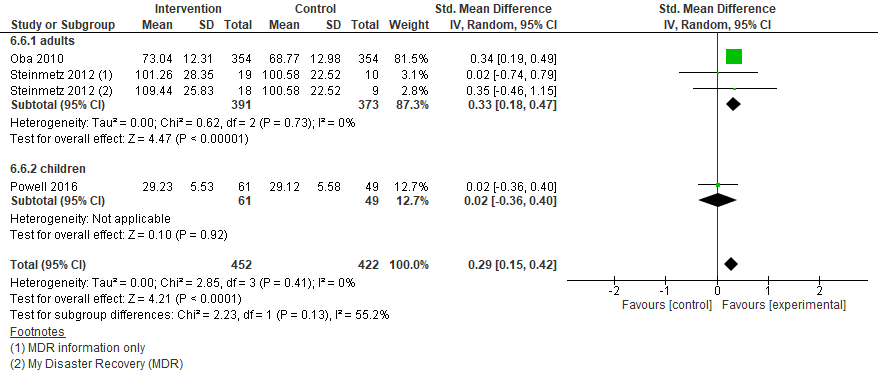


Figure 58: Wellbeing in intervention-control studies (subgroups by extreme weather events)


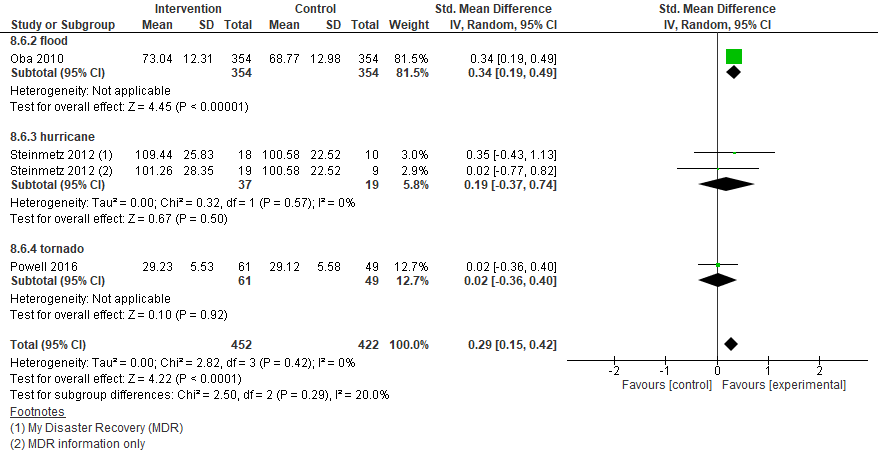


Figure 59: Wellbeing in before-after studies


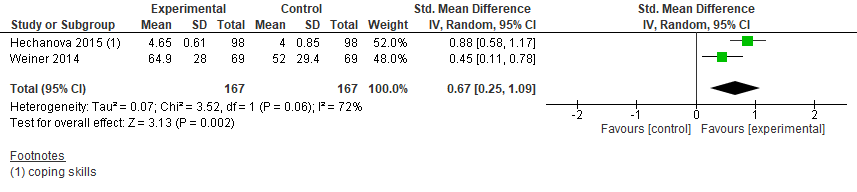


Figure 60: Wellbeing in before-after studies (subgroups by scales)


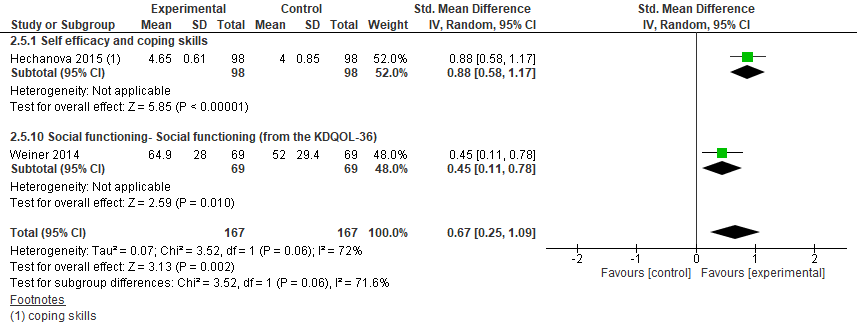


Figure 61: Wellbeing in before-after studies (subgroups by SES)


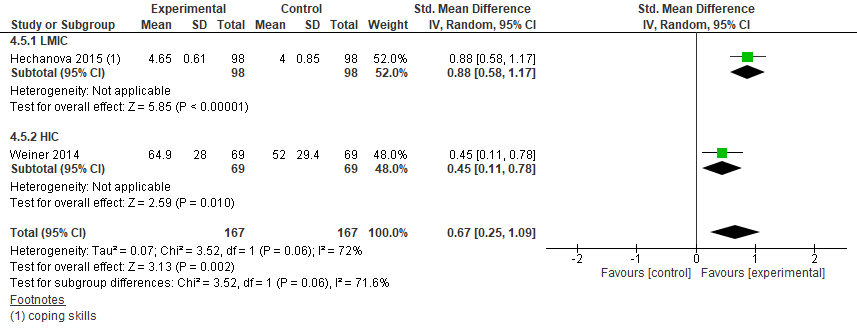


Figure 62: Wellbeing in before-after studies (subgroups by age)


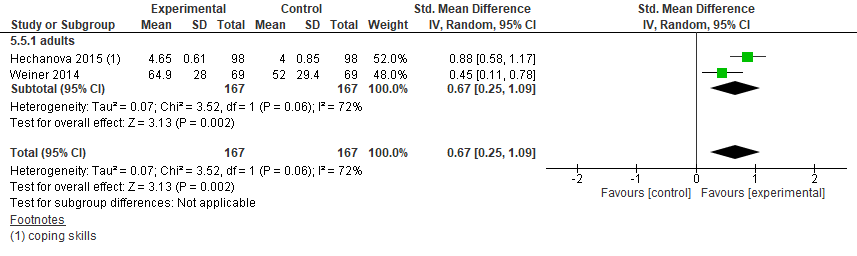


Figure 63: Wellbeing in before-after studies (subgroups by extreme weather events)


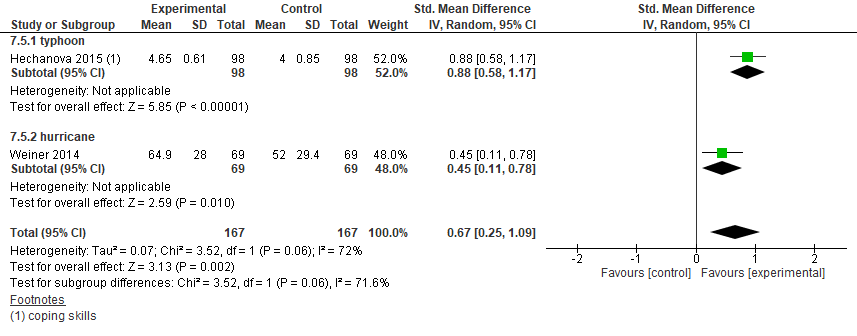

Supplement: Supplementary file 1 [file bmjgh-11-5-s001.docx]
