# Supplementary material for: Systematic review and meta-analysis of interventions for mental health in extreme weather events
Source: BMJ Glob Health. 2026 May 28;11(5):e020407. doi: 10.1136/bmjgh-2025-020407 (PMC13223657; doi:10.1136/bmjgh-2025-020407)
Supplement: Supplementary file 2 [file bmjgh-11-5-s002.docx]

### BMJ Global Health Author Reflexivity Statement

Adapted from Morton, B., Vercueil, A., Masekela, R., Heinz, E., Reimer, L., Saleh, S., Kalinga, C., Seekles, M., Biccard, B., Chakaya, J., Abimbola, S., Obasi, A. and Oriyo, N. (2022), Consensus statement on measures to promote equitable authorship in the publication of research from international partnerships. Anaesthesia, 77: 264-276. <https://doi.org/10.1111/anae.15597>

| **Study conceptualisation** | |
| --- | --- |
| 1. How does this study address local research and policy priorities? | This is a systematic review and the findings are relevant for both the developed and developing countries |
| 1. How were local researchers involved in study design? | This is a global systematic review and most of the authors are from developing countries. |
| **Research management** | |
| 1. How has funding been used to support the local research team(s)? | The fundings supported staff and project activities |
| **Data acquisition and analysis** | |
| 1. How are research staff who conducted data collection acknowledged? | The data extractors are the authors on this. |
| 1. How have members of the research partnership been provided with access to study data? | The data is from existing published papers which are available online |
| 1. How were data used to develop analytical skills within the partnership? | This systematic review was led by and conducted in a developing country and the researchers are the experts in systematic review. |
| **Data interpretation** | |
| 1. How have research partners collaborated in interpreting study data? | Through regular meetings and consultations |
| **Drafting and revising for intellectual content** | |
| 1. How were research partners supported to develop writing skills? | This systematic review was led by and conducted in a developing country and the researchers are the experts in systematic review and writing. |
| 1. How will research products be shared to address local needs? | The findings would be disseminated via local and international conference publications and through local and social media. |
| **Authorship** | |
| 1. How is the leadership, contribution and ownership of this work by LMIC researchers recognised within the authorship? | They are the lead authors. |
| 1. How have early career researchers across the partnership been included within the authorship team? | Three early career researchers who were engaged in this systematic review are the authors on it. |
| 1. How has gender balance been addressed within the authorship? | There is a good gender balance as 40% of the authors are females. |
| **Training** | |
| 1. How has the project contributed to training of LMIC researchers? | This systematic review was led by and conducted in a developing country and the researchers are the experts in systematic review. |
| **Infrastructure** | |
| 1. How has the project contributed to improvements in local infrastructure? | N/A |
| **Governance** | |
| 1. What safeguarding procedures were used to protect local study participants and researchers? | N/A |
